# Modeling freshwater inflow to a tropical estuary using stochastic and neural-network approaches under contrasting hydroclimatic conditions in Brazil

**DOI:** 10.1007/s10661-026-16034-1

**Published:** 2026-10-17

**Authors:** Luísa Maria de Souza Viana, Eloá Corrêa Lessa Tostes, Wendel Dias Constantino, Felipe Henrique Rossi Luze, Marcos Sarmet Moreira de Barros Salomão, Taíse Bonfim de Jesus, Carlos Eduardo Veiga de Carvalho

**Affiliations:** 1 Programa de Pós-Graduação Em Ecologia E Recursos Naturais, Laboratório de Ciências Ambientais, Centro de Biociências E Biotecnologia, Universidade Estadual Do Norte Fluminense Darcy Ribeiro, Av. Alberto Lamego, 2000 – Parque Califórnia, CEP: 28013-602 Campos Dos Goytacazes, Rio de Janeiro, Brazil; 2 Departamento de Ciências Exatas, Universidade Estadual de Feira de Santana, Feira de Santana, Bahia, Brazil; 3 Laboratório de Ciências Químicas, Centro de Ciência E Tecnologia, Universidade Estadual Do Norte Fluminense Darcy Ribeiro, Av. Alberto Lamego, 2000 – Parque Califórnia –, CEP: 28013-602 Campos Dos Goytacazes, Rio de Janeiro, Brazil

**Keywords:** Data-scarce catchments, Streamflow reconstruction, Discontinued gauging stations, Hydroclimatic variability, Coastal basin monitoring, Time-series modeling

## Abstract

Freshwater inflow regulates estuarine circulation, salinity gradients, sediment transport, and biogeochemical fluxes, yet long-term discharge records remain scarce in many tropical coastal basins. We compared linear regression, stochastic time-series models (ARMA, ARIMA, and SARIMA), and multilayer perceptron (MLP) neural networks for reconstructing monthly discharge at the Ituberá gauging station, upstream of the Serinhaém estuary (southern Bahia, Brazil), using records from 1966 to 2019. No model family was uniformly superior across rolling-origin selection and independent validation. Predictor-matched comparisons showed no general advantage of MLPs over linear formulations, and extending calibration through 2011 did not consistently improve post-2012 performance. Mean monthly discharge was approximately 15% lower after 2012, while changes in variance and the general seasonal pattern were not detected and evidence for altered temporal dependence remained inconclusive. One-step reconstructions transferred more consistently to held-out data than recursive and longer-horizon predictions, which showed error accumulation. The reconstructed series provides an upstream freshwater boundary indicator for future estuarine modeling, while ecological consequences require explicit hydrodynamic and biogeochemical simulation.

**Supplementary Information:**

The online version contains supplementary material available at https://doi.org/10.1007/s10661-026-16034-1.

## Introduction

Freshwater inflow is a fundamental driver of estuarine structure and functioning, controlling salinity gradients, stratification, circulation, sediment transport, residence times, and the delivery and fate of nutrients, organic matter, and contaminants from watersheds to coastal zones (Alongi, 2008; Broadley et al., 2022; de Souza Viana et al., 2024; Geyer & MacCready, 2014; Lebreton et al., 2016). This control is particularly important in tropical estuaries, where river discharge is sensitive to climate variability, extreme rainfall, and prolonged droughts. At monthly to seasonal timescales, hydroclimatic fluctuations modify the balance between riverine advection and tidal mixing, potentially affecting exchange flows, salinity intrusion, and biogeochemical cycling (Alongi, 2014; Bronstert et al., 2002). Reliable monthly discharge estimates are therefore important for understanding estuarine variability and providing boundary conditions for hydrodynamic and biogeochemical assessments. In the absence of site-specific hydrodynamic simulations, however, the estuarine consequences of discharge variability can only be interpreted conceptually.

Hydrological monitoring networks in many tropical regions remain sparse or discontinuous, limiting long-term assessments of river discharge and freshwater delivery to estuaries. This constraint is particularly relevant in developing regions, where continuous records are unavailable for many small and medium-sized coastal catchments (Adamowski et al., 2012; Melo et al., 2020; Viana et al., 2021). In Brazil, limitations in the spatial and temporal coverage of in situ hydrological observations, including those from coastal catchments, constrain assessments of hydroclimatic variability and its potential influence on freshwater delivery to estuarine environments (Melo et al., 2020; Viana et al., 2021). Reconstructing discontinued or incomplete discharge records can consequently support retrospective assessments and provide a basis for future environmental modeling. In data-limited basins, hydrological modeling can also be supported by complementary gridded meteorological datasets, which provide additional hydroclimatic information where in situ observations are sparse (Viana et al., 2021).

Linear regression and stochastic time-series approaches, including autoregressive moving average (ARMA), autoregressive integrated moving average (ARIMA), and seasonal ARIMA (SARIMA), remain widely used because of their transparency and relatively low data requirements (Adamowski & Karapataki, 2010; Box et al., 2015; Zhang, 2003). These models can represent linear predictor–response relationships, serial dependence, and seasonal patterns. Their performance may nevertheless deteriorate when hydrological responses are nonlinear or when relationships learned during calibration do not transfer to hydroclimatically contrasting periods (Milly et al., 2008; Nourani & Komasi, 2013; Valipour et al., 2012). Comparisons with nonlinear approaches should therefore employ common evaluation periods and clearly distinguish improvements arising from model structure from those arising from additional predictor information.

Machine learning methods provide a complementary framework for representing potentially nonlinear relationships between climatic predictors and hydrological responses. Feedforward multilayer perceptrons (MLP) have been widely applied to streamflow modeling, although their performance depends on data availability, predictor selection, network architecture, and the hydroclimatic setting (Ghorbani et al., 2016; Maier & Dandy, 2000; Mehr et al., 2015; Mosavi et al., 2018). Unlike recurrent architectures, MLPs do not contain an internal recurrent state, and temporal information must be supplied explicitly through lagged predictors (Maier et al., 2010; Zounemat-Kermani et al., 2020). Long short-term memory (LSTM) and gated recurrent unit (GRU) networks can learn sequential dependencies through internal memory mechanisms, but their principal advantages are generally better exploited using large and hydrologically diverse training datasets (Kratzert et al., 2018, 2024). Given the monthly resolution and single-basin length of the available record, a shallow MLP was selected as a comparatively parsimonious nonlinear alternative rather than on the assumption that it is inherently superior to recurrent architectures.

Model transferability is particularly relevant when the evaluation period includes prolonged hydroclimatic extremes. Northeast Brazil experienced a severe and persistent drought beginning in 2012, characterized by widespread precipitation deficits and sustained hydrological stress (Bruno et al., 2024; Marengo et al., 2017). Such conditions may affect discharge magnitude and variability and expose model limitations that are less evident during calibration. The post-2012 period was therefore adopted as a drought-focused evaluation interval. Because formal tests did not establish an unequivocal drought-related change point or monotonic trend in the available discharge record, this interval is treated as hydroclimatically contrasting rather than as a formally demonstrated non-stationary regime. Although statistical and machine learning approaches are widely applied to streamflow prediction, evidence specifically evaluating the temporal transferability of discharge reconstructions across prolonged drought conditions in tropical coastal basins remains limited (Maier et al., 2010; Melo et al., 2020; Zounemat-Kermani et al., 2020).

The Serinhaém River basin in southern Bahia illustrates these monitoring and modeling challenges. The river drains into an ecologically important estuary within a protected coastal region of the Atlantic Forest biome, but the available discharge record from the Ituberá gauging station ends in 2019 (ANA, 2026). Because the station is located approximately 21.21 km upstream from the estuary mouth and represents only the upstream portion of the contributing drainage area, its measurements do not include intervening tributaries, abstractions, or other downstream gains and losses. Gauged discharge is therefore treated as an indicator of freshwater delivery to the Serinhaém estuary rather than as a direct measurement of total freshwater inflow at its mouth.

This study evaluates the predictive performance and temporal transferability of multiple linear regression, ARMA, ARIMA, SARIMA, and univariate and precipitation-informed MLP models for reconstructing monthly discharge at the Ituberá station. T1 uses calibration data through 2005 and evaluates the subsequent record, whereas T2 uses the longer and more recent calibration record through 2011 and focuses on the post-2012 period. The models are assessed through held-out one-step predictions, rolling-origin validation, common-period comparisons, and retrospective prediction horizons of 1, 3, 6, 12, and 24 months. Completely recursive trajectories are examined separately to evaluate the limitations of long uninterrupted reconstructions. Here, reconstruction denotes the retrospective estimation of monthly discharge during observed but withheld periods used to simulate the discontinuation of monitoring. It does not refer to operational forecasting or to the direct estimation of total freshwater inflow at the estuary mouth. The term *estimation* is used only in its general statistical sense, while *hindcasting* and *operational forecasting* are not used because the analysis reconstructs withheld historical observations rather than predicting genuinely unknown future conditions. The motivating application is the extension of a discharge record after regular observations cease, rather than the imputation of isolated missing observations within the historical series. Based on these objectives and definitions, we tested the following hypotheses:

- H1: Under common evaluation periods and comparable information domains, MLP configurations produce lower monthly discharge errors than their conventional counterparts, with the univariate MLP compared against discharge-based stochastic models and the precipitation-informed MLP compared against linear regression.
- H2: Models calibrated using the longer and more recent record immediately preceding the drought-focused period (T2) produce lower errors during the common post-2012 evaluation period than models calibrated using the earlier historical record (T1).
- H3: The post-2012 discharge series differs from the preceding record in its magnitude, variability, seasonality, or temporal autocorrelation, potentially affecting the temporal transferability of models calibrated using earlier observations.

## Materials and methods

### Study area and datasets

**Fig. 1:**
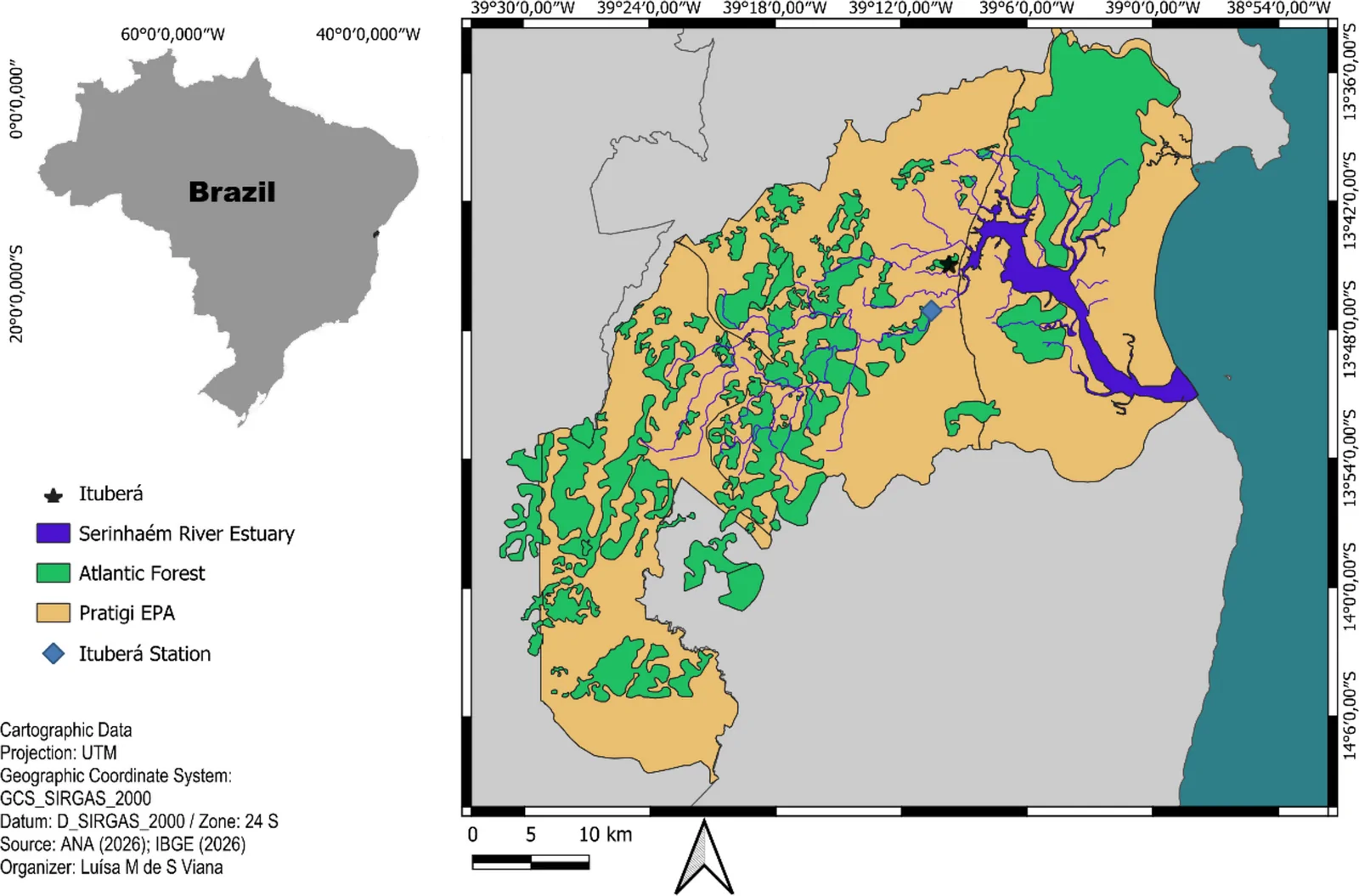
Location of the Serinhaém River basin and estuary in southern Bahia, Brazil, including the ANA gauging station used for monthly discharge records and the connection with Camamu Bay

This study focused on the Serinhaém River basin in southern Bahia, Brazil, which drains into the Serinhaém estuary and Camamu Bay and is located within the Pratigi Environmental Protection Area (Fig. 1). The complete drainage basin covers approximately 473.68 km^2^ (MMA, 2004) and lies within the Atlantic Forest biome. The region has a tropical humid climate (Köppen–Geiger Af), with mean annual precipitation of approximately 2,095 mm and a mean annual temperature of 24.6 °C (de Amorim et al., 2015). Rainfall occurs throughout the year without a marked dry season, although July is generally the wettest month and September the driest (Carneiro et al., 2021).

Monthly river discharge was obtained from the Ituberá gauging station (code 51940000; 13.7853° S, 39.1758° W), located on the Cachoeira Grande River in the municipality of Ituberá. The station is under the responsibility of the Brazilian National Water and Basic Sanitation Agency (ANA), is operated by the Geological Survey of Brazil (SGB-CPRM), and represents a gauged drainage area of 263 km^2^. The station is located approximately 21.21 km upstream from the estuary mouth. Consequently, its record does not include intervening tributaries, water abstractions, or other hydrological gains and losses occurring along the downstream reach. Station discharge was therefore interpreted as an indicator of freshwater delivery toward the Serinhaém estuary rather than as a direct measurement of total freshwater inflow at its mouth.

Monthly precipitation was obtained from a rain gauge located in Camamu, Bahia (13.93° S, 39.16° W). The precipitation record originally extended from 1966 to 2020, but only observations through April 2019 were retained to match the discharge period. All data were obtained at, or aggregated to, monthly resolution. No daily or sub-monthly observations were included, and all predictors, lags, prediction horizons, and performance metrics refer to monthly time steps.

The resulting common temporal grid extended from January 1966 to April 2019 and contained 640 months. Of these, 634 months had observed discharge, 631 had observed precipitation, and 628 had observations for both variables. Missing months were retained in their original positions to preserve the monthly spacing of the series and the correct construction of lagged predictors. The data sources, station characteristics, temporal coverage, and missing observations are documented in Table S1 and in the data-audit files.

### Missing data and predictor construction

Missing discharge and precipitation observations were retained as missing values and were not imputed. Therefore, no estimated values were introduced into model calibration, predictor construction, or performance evaluation. Lagged variables associated with a missing observation were also retained as missing. Model fitting and prediction were performed using the observations for which the response and all predictors required by the corresponding model configuration were available.

Discharge and precipitation observations that were originally missing were flagged throughout the analytical workflow. Performance metrics were calculated only for months with observed discharge and a finite model prediction. Consequently, the number of evaluated months differed slightly among model families, particularly for precipitation-informed models, because missing precipitation observations propagated to their corresponding lagged predictors. Like-for-like model comparisons were conducted only over dates shared by the models included in each contrast.

Lagged predictors were constructed for discharge, denoted by *Q*, and precipitation, denoted by *P*. Candidate configurations included recent monthly lags and, when applicable, a 12-month lag representing annual temporal dependence. Univariate configurations used lagged discharge exclusively, whereas precipitation-informed configurations combined lagged discharge and precipitation. The complete candidate sets and predictors selected for T1 and T2 are reported in Table S2.

Each modeling sample corresponded to a target month t, with discharge ${Q}_{t}$ as the response and the required antecedent values ${Q}_{t-k}$ and, for precipitation-informed configurations, ${P}_{t-k}$ as predictors. A sample was used only when the target discharge and all lagged predictors required by that configuration were available.

For MLP fitting, predictors and response values were standardized using z-scores. The mean and standard deviation were estimated exclusively from the applicable calibration sample or from the training portion of each rolling-origin fold and were then applied to the corresponding prediction sample. Predicted values were subsequently transformed back to m^3^ s⁻^1^ before evaluation. Multiple linear regression and stochastic time-series models were fitted in physical units.

### Temporal partitioning

Two temporal configurations were used to examine model performance under different calibration and hydroclimatic settings. In T1, January 1966–December 2005 was used for calibration and January 2006–April 2019 for validation. This partition contained 480 calibration months, of which 476 had observed discharge, and 160 validation months, of which 158 had observed discharge.

In T2, January 1966–December 2011 was used for calibration and January 2012–April 2019 for validation. This partition contained 552 calibration months, of which 547 had observed discharge, and 88 validation months, of which 87 had observed discharge. T2 was treated as a drought-focused evaluation configuration because its validation period coincided with the prolonged drought affecting Northeast Brazil after 2012. However, this period was not assumed a priori to constitute a formally established structural break or non-stationary regime.

T1 and T2 differed in calibration length, calibration recency, and validation period. Metrics calculated over their respective full validation periods were therefore used to describe model performance within each configuration but were not interpreted as direct evidence of an effect of calibration updating (Figure S2). To provide a like-for-like comparison, predictions from both configurations were also evaluated over their common period, January 2012–April 2019. Within each model family, T1 and T2 predictions were compared using the same observed months.

### Rolling-origin cross-validation and model selection

Candidate models and hyperparameter configurations were selected using a common rolling-origin cross-validation framework that preserved the temporal ordering of the observations. The procedure was applied to all model families, including multiple linear regression, ARMA, ARIMA, SARIMA, and the univariate and precipitation-informed MLP configurations.

For each temporal configuration, an expanding training window was used, with a minimum initial training period of 20 years. Candidate forecast origins were defined within the final 120 months of the corresponding calibration period and advanced at three-month intervals. At each origin, the model was fitted using only information available up to that date and was evaluated through a one-month-ahead prediction. This produced a maximum of 40 candidate folds for T1 and 39 for T2. Folds lacking an observed discharge target, the predictor history required by a candidate configuration, or a successfully converged model fit were excluded from the corresponding cross-validation summary. The number and proportion of successful folds were recorded for every candidate model.

For the regression and MLP models, standardization parameters were re-estimated within the training portion of every fold. Stochastic models were fitted in physical discharge units. Candidate configurations were ranked primarily according to their mean cross-validated RMSE, with cross-validated MAE and the proportion of successful folds used as complementary criteria. The external validation periods were not used for predictor selection, stochastic-model selection, or MLP hyperparameter tuning.

After selection, each model was refitted using the complete calibration sample available for its temporal configuration and evaluated over the corresponding held-out validation period. Fold-level cross-validation results and the selected models are provided in the Supplementary Material.

### Seasonal-naive benchmark

A seasonal-naive model was included as a minimum-performance benchmark. For each target month, this model used the discharge observed in the same calendar month of the preceding year:

$$\widehat{Q}t=Qt-12.$$

This benchmark represents the predictive information contained in the historical annual cycle without estimating additional model parameters. Candidate models were expected to improve upon this reference, particularly according to the MASE and RSR criteria.

### Stochastic time-series models

ARMA, ARIMA, and SARIMA models were developed following the Box–Jenkins framework (Box et al., 2015). Candidate autoregressive, moving-average, differencing, and seasonal orders were informed by autocorrelation and partial-autocorrelation diagnostics and subsequently evaluated through the common rolling-origin procedure.

ARMA models represented nondifferenced autoregressive and moving-average dependence. ARIMA models allowed nonseasonal differencing, whereas SARIMA models additionally included seasonal autoregressive, differencing, or moving-average components with a 12-month periodicity. Candidate formulations were fitted using the calibration data available at each origin. AICc and residual diagnostics were used to assess model adequacy, whereas predictive selection was based on rolling-origin performance rather than on the external validation period.

The selected stochastic specifications were ARMA(1,0,2), ARIMA(1,1,3), and SARIMA(2,1,2)(2,0,0)[12] for T1, and ARMA(1,0,2), ARIMA(2,1,4), and SARIMA(3,1,2)(1,0,1)[12] for T2. Complete candidate results are reported in the Supplementary Material.

### Multiple linear regression

Multiple linear regression was implemented as a transparent reference for linear relationships between monthly discharge and lagged hydrological predictors. Candidate models differed in their combinations of lagged discharge and precipitation and were evaluated using the same rolling-origin cross-validation structure adopted for the other model families.

The selected T1 model (M6) included Q1, Q2, Q3, P1, and P2, whereas the selected T2 model (M1) included Q1 and P1. The regression models were fitted in physical units using complete cases for the variables included in each configuration. In addition to these independently selected models, complementary predictor-matched analyses fitted linear regression and MLP models using identical input variables to distinguish differences attributable to model structure from those attributable to predictor information.

### Multilayer perceptron models

Feedforward multilayer perceptron neural networks were used to represent potentially nonlinear relationships between lagged predictors and monthly discharge. Two information domains were considered. The univariate MLP used lagged discharge exclusively, whereas the precipitation-informed MLP combined lagged discharge and precipitation. Comparisons with conventional models were conducted over common prediction dates to reduce differences caused by missing predictors or unequal evaluation samples.

All primary MLPs contained an input layer, one hidden layer, and a single linear output unit. Models were fitted using the nnet implementation, with a logistic sigmoid activation function in the hidden layer and BFGS optimization. The models were trained using a squared-error objective with L2 weight-decay regularization controlled by the decay parameter. Alternative training loss functions were not evaluated. No validation-based early stopping was used. Optimization proceeded until the BFGS convergence criterion was reached or the maximum of 1,000 iterations was attained. Therefore, no separate validation subset, stopping epoch, or training and validation loss curves were generated for the primary MLP models.

Candidate configurations differed in predictor set, number of hidden units (1, 2, 3, 4, 5, 6, 8, and 10), and weight-decay value (0, 0.0001, 0.001, and 0.01). Candidate configurations were evaluated using the same rolling-origin cross-validation framework applied to the conventional models. Predictors and response values were standardized using parameters estimated exclusively from the corresponding training data, and predictions were subsequently transformed back to m^3^ s⁻^1^. Three fixed-seed random initializations were used for each candidate MLP within each rolling-origin fold. Model selection was based primarily on cross-validated RMSE, with cross-validated MAE and the proportion of successful folds used as complementary criteria. The independent validation periods were not used for hyperparameter tuning or model selection.

The final selected networks were fitted using 20 fixed-seed random initializations. For each target month, predictions from the successfully fitted networks were back-transformed to physical units and averaged. This arithmetic mean constituted the MLP ensemble prediction used in the figures and in the calculation of all performance metrics. Sensitivity to random initialization was additionally quantified by evaluating the 20 final fits separately. The coefficient of variation of independent-validation RMSE ranged from 1.83 to 27.3% across the selected MLP configurations, whereas the corresponding coefficient of variation of MAE ranged from 1.56 to 11.5%. Sensitivity was therefore model-dependent, and averaging across the 20 fits reduced dependence of the final prediction on any single initialization. The ensemble predictions reproduced the validation performance reported for the selected MLP models.

Because the MLPs were fitted directly to explicitly lagged monthly predictors, no frequency or differencing parameter was used in the MLP specification. The primary hyperparameter grid is summarized in Table S2, and candidate-model results are reported in Tables S6 and S7.

The selected T1 univariate configuration was U3, using Q1, Q2, and Q3 with eight hidden units and no weight decay, resulting in a 3–8-1 architecture. The selected T2 univariate configuration was U5, using Q1, Q2, Q3, and Q12 with four hidden units and a decay value of 0.01, resulting in a 4-4-1 architecture. For the precipitation-informed MLP, the selected T1 configuration was M3, using Q1, Q2, P1, and P2 with four hidden units and a decay value of 0.01, resulting in a 4-4-1 architecture. The selected T2 configuration was M4, using Q1, Q2, Q12, P1, and P2 with ten hidden units and a decay value of 0.001, resulting in a 5–10−1 architecture.

Because the feedforward MLP has no intrinsic recurrent or seasonal state, temporal and seasonal information was supplied explicitly through lagged predictors. The selected configurations used recent discharge lags and, where selected, Q12 to represent annual-lag information. As a sensitivity analysis, univariate MLPs using continuous 12- and 24-month discharge windows were also evaluated using the same rolling-origin cross-validation procedure.

Additional exploratory sensitivity analyses examined whether MLP performance depended on activation function and optimization settings. Logistic sigmoid, hyperbolic tangent (tanh), and rectified linear unit (ReLU) hidden-layer activations were compared using a custom single-hidden-layer implementation, with hidden-layer size and weight decay re-optimized within each activation using rolling-origin cross-validation. Learning rates of 0.0001, 0.0005, 0.001, 0.005, 0.01, 0.05, and 0.1 were evaluated, with 0.01 used as the reference value and a maximum training budget of 2000 epochs. Because the custom logistic BFGS implementation did not exactly reproduce predictions obtained with the primary nnet implementation, these analyses were treated strictly as sensitivity checks and were not used to reselect the primary MLP configurations. A separate custom backpropagation analysis evaluated sensitivity to learning rate under a maximum training budget of 2000 epochs. These optimization experiments were likewise considered exploratory and did not alter primary model selection.

### One-step, recursive, and multi-horizon evaluation

The primary held-out evaluation was based on sequential one-step-ahead predictions. For each validation month, observed discharge values from preceding months were used when required as lagged predictors. This evaluation quantified the ability of each fitted model to estimate the following monthly observation while preserving a realistic temporal ordering. For precipitation-informed MLPs, the corresponding observed historical precipitation values were supplied at the required lags; precipitation was not recursively predicted.

Completely recursive trajectories were evaluated separately. Under this configuration, after the beginning of the validation period, model-generated discharge estimates were returned to the predictor history and used instead of subsequently observed discharge values. This analysis was used to examine error propagation during long uninterrupted reconstructions and was not treated as the primary estimate of monthly predictive performance.

Retrospective prediction performance was also evaluated at horizons of 1, 3, 6, 12, and 24 months. The multi-horizon analysis quantified how errors changed as the distance from the last observed discharge value increased. Because the intended application is retrospective reconstruction rather than operational forecasting, the historical precipitation record was considered available where required by precipitation-informed models. The number of valid predictions was recorded separately for every model and horizon because missing predictors and increasing forecast horizons reduced the available sample. Multi-horizon and completely recursive results are presented as supplementary sensitivity analyses.

### Predictive uncertainty

Predictive uncertainty for the selected MLP configurations was evaluated using a nonparametric moving-block residual bootstrap restricted entirely to calibration information. Pseudo-out-of-sample one-step residuals were first generated within each calibration period using an expanding-window procedure, with each target month predicted using only observations available before that month. Residuals were calculated as observed discharge minus the corresponding ensemble-mean MLP prediction. Validation-period observations and errors were not used to construct the uncertainty intervals.

Successful calibration residuals were centered and organized into overlapping contiguous 12-month blocks to preserve short-term temporal dependence and annual structure. Each block constituted the resampling unit. Blocks were sampled with replacement and concatenated to generate 2000 bootstrap residual series for each selected MLP configuration. The resampled residuals were added to the corresponding independent one-step ensemble-mean predictions, and simulated discharge values were constrained to be non-negative. For each validation month, the 2.5th and 97.5th percentiles of the resulting bootstrap distribution defined the empirical 95% predictive interval.

Point-performance metrics were calculated once by comparing observed discharge with the ensemble-mean prediction and were not calculated separately for each bootstrap realization. The bootstrap distributions were used to construct the predictive intervals and to calculate empirical coverage and interval-width diagnostics.

These intervals quantify residual-based predictive uncertainty conditional on the selected model, predictors, hyperparameters, calibration residuals, block length, and bootstrap procedure. They do not incorporate uncertainty associated with model selection, alternative network architectures, predictor selection, measurement error, or structural hydrological uncertainty.

### Performance metrics

Model performance was evaluated using complementary measures of absolute error, scale-normalized error, overall predictive skill, systematic bias, association, and representation of discharge variability. Primary error metrics were root mean squared error (RMSE), mean absolute error (MAE), the ratio of RMSE to the standard deviation of observed discharge (RSR), mean absolute scaled error (MASE), and mean prediction bias.

$$RMSE=\sqrt{\frac{1}{n}\sum_{t=1}^{n}{\left({Q}_{t}^{\left(obs\right)}-{Q}_{t}^{\left(pred\right)}\right)}^{2}}$$

$$MAE=\frac{1}{n}\sum_{t=1}^{n}\left|{Q}_{t}^{\left(obs\right)}-{Q}_{t}^{\left(pred\right)}\right|$$

$$RSR=\frac{RMSE}{SD\left({Q}^{obs}\right)}$$

$$MASE=\frac{\left.\frac{1}{n}\sum_{t=1}^{n}\left|{Q}_{t}^{\left(obs\right)}-{Q}_{t}^{\left(pred\right)}\right|\right)}{\left.\frac{1}{T-12}\sum_{t=13}^{T}\left|{Q}_{t}^{\left(cal\right)}-{Q}_{t-12}^{\left(cal\right)}\right|\right)}$$

$$Bias=\frac{1}{n}\sum_{t=1}^{n}\left({Q}_{t}^{\left(pred\right)}-{Q}_{t}^{\left(obs\right)}\right)$$

where ${Q}_{t}^{\left(obs\right)}$ and ${Q}_{t}^{\left(pred\right)}$ are the observed and predicted discharge values for month *t*, respectively; *n* is the number of valid observed–predicted pairs; ${Q}_{t}^{\left(cal\right)}$ denotes discharge in the calibration period; and *T* is the number of monthly calibration observations used to calculate the seasonal scaling term.

To avoid ambiguity in the definition of normalized RMSE, scale-normalized RMSE was reported as the RMSE–standard deviation ratio (RSR). For each model and evaluation strategy, the standard deviation in the RSR denominator was calculated from the same finite observed discharge values used to calculate the corresponding RMSE. In matched-date comparisons, RSR was therefore calculated using the same observed months for both models or temporal configurations.

Positive Bias values indicate average overprediction, whereas negative values indicate average underprediction. MASE scales model MAE relative to the in-sample error of the seasonal-naive benchmark calculated from the corresponding calibration series using a 12-month seasonal lag (Hyndman & Koehler, 2006). Within a fixed evaluation sample, lower RMSE, MAE, RSR, and MASE values indicate smaller prediction error, while Bias values closer to zero indicate lower systematic error. Because RSR and MASE depend on their scaling denominators, they were not interpreted alone as evidence of improved predictive skill across periods with different discharge variability. A MASE value below 1 indicates lower MAE than the seasonal-naive reference for the corresponding calibration-based scaling.

**Fig. 2:**
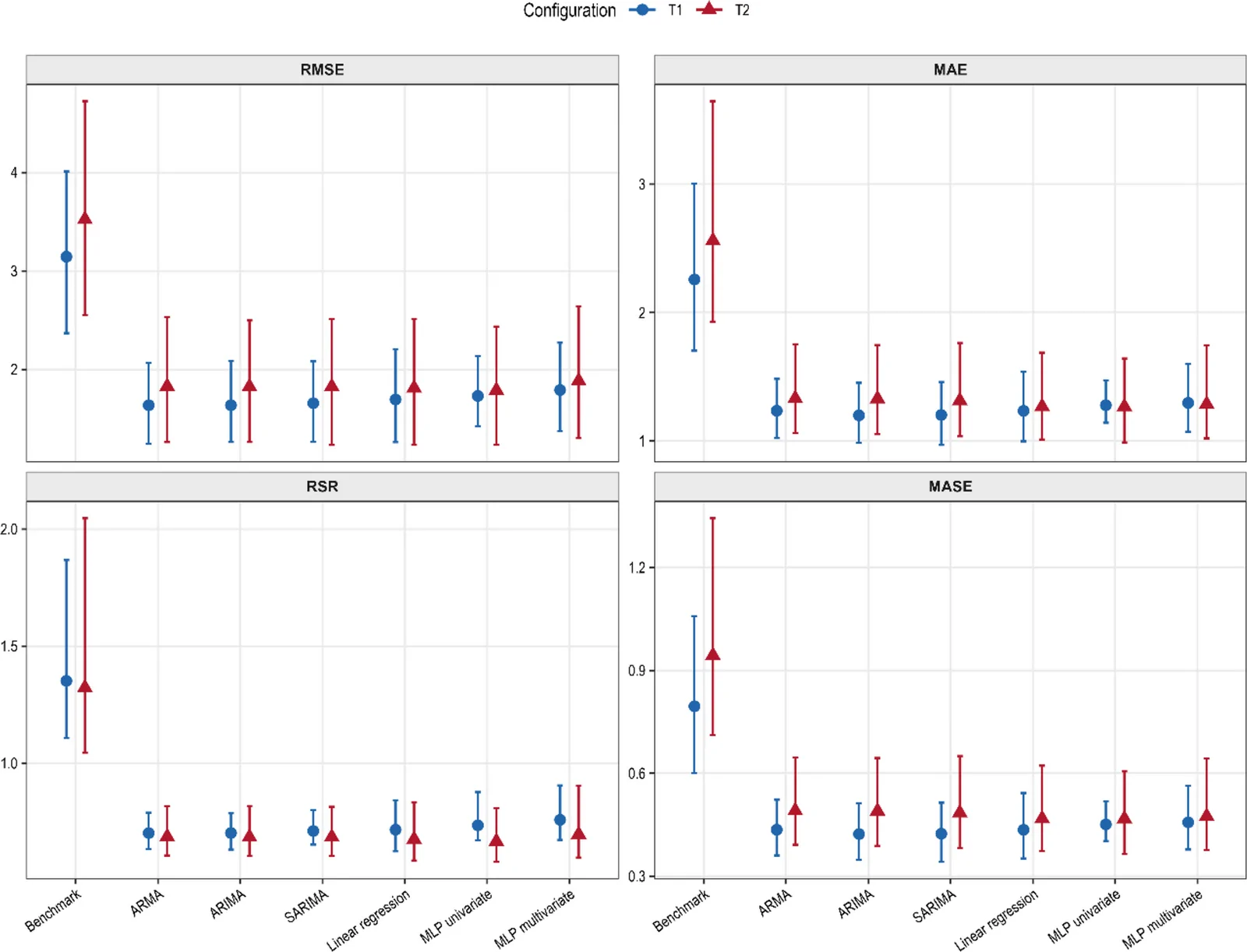
Comparative one-step validation performance of the selected predictive models under the T1 and T2 temporal configurations. Panels show root mean square error (RMSE), mean absolute error (MAE), the ratio of RMSE to the standard deviation of observed discharge (RSR), and mean absolute scaled error (MASE). Points represent the metrics calculated from the independent validation predictions, and error bars represent 95% confidence intervals obtained using a 12-month moving-block bootstrap with 2,000 replicates. Lower values indicate better predictive performance. Blue circles represent T1 and red triangles represent T2. Because T1 and T2 involved different validation periods and numbers of valid predictions, cross-configuration comparisons are descriptive

Uncertainty in the independent-validation RMSE, MAE, RSR, and MASE estimates shown in Fig. 2 was quantified using a moving-block bootstrap of temporally ordered observed–predicted pairs. Contiguous 12-month blocks were resampled with replacement in 2,000 replicates, and the 2.5th and 97.5th percentiles of the resulting metric distributions were used as 95% confidence interval.

Complementary metrics were used to characterize different and nonredundant aspects of model performance (Gupta et al., 1998), including Nash–Sutcliffe efficiency (NSE), the modified Kling–Gupta efficiency (KGE2012; Kling et al., 2012), percent bias (PBIAS), the squared Pearson correlation coefficient (*R*^2^), and relative variance error. Relative variance error was calculated as $\left[Var\left({Q}\right)-Var\left(Q\right)\right]/Var\left(Q\right)$; values close to zero indicate similar observed and predicted variability, negative values indicate underestimation of discharge variance, and positive values indicate overestimation. These complementary metrics were used particularly to assess whether conclusions based on absolute error were consistent with scale-normalized performance and representation of observed discharge variability.

All metrics were calculated using only finite observed–predicted pairs. For direct comparisons involving two or more models, metrics were recalculated using only the intersection of dates for which valid observations and predictions were available for all models included in the corresponding contrast.

Flow-dependent error structure was additionally examined by dividing observed discharge into low, intermediate, and high-flow classes using terciles defined separately within each temporal configuration and evaluation stage. For the selected MLP configurations, RMSE, MAE, mean Bias, and the fraction of underpredicted observations were calculated for each flow class using both pseudo-out-of-sample calibration predictions and independent validation predictions. This analysis was used to determine whether systematic overprediction of low flows or underprediction of high flows was already present during calibration or emerged only during independent validation (Table S12).

### Statistical comparison of predictive errors

Differences in one-step predictive performance were evaluated using the Diebold–Mariano test applied to absolute-error loss differentials, with a one-month prediction horizon and a two-sided alternative (Diebold & Mariano, 1995). Tests used the autocovariance-based long-run variance estimator and the Harvey, Leybourne, and Newbold small-sample correction implemented in *forecast::dm.test* (Harvey et al., 1997). The null hypothesis was equal predictive accuracy between the two models. Tests were conducted using matched prediction dates and therefore compared errors generated for the same observed discharge values.

For H1, two complementary comparison sets were performed. First, the selected univariate MLP was compared separately with the selected ARMA, ARIMA, and SARIMA models, whereas the selected precipitation-informed MLP was compared with multiple linear regression. These comparisons were conducted separately under T1 and T2 using only dates shared by the models involved in each contrast. The eight resulting *p*-values were adjusted using the Benjamini–Hochberg procedure (Benjamini & Hochberg, 1995). Second, to distinguish nonlinear model structure from differences in predictor information, linear regression and MLP models were compared using identical predictor sets and validation dates. Predictor-matched analyses were conducted for a discharge-only information domain (Q1, Q2, and Q3) and a discharge-plus-precipitation domain (Q1, Q2, Q3, P1, and P2). The four predictor-matched p-values were adjusted separately using the Benjamini–Hochberg procedure. MLP hyperparameters for these complementary comparisons were selected using calibration-only rolling-origin cross-validation without using the independent validation period.

For H2, T1 and T2 predictions were compared within each model family over their common post-2012 prediction dates, ensuring that both temporal configurations were evaluated against the same observed discharge values. Absolute-error comparisons based on RMSE and MAE were complemented by RSR, MASE, and relative variance error over the same common evaluation period to determine whether conclusions were consistent across absolute accuracy, scale-normalized performance, and representation of discharge variability. Because several model-family contrasts were evaluated simultaneously, inferential *p*-values were adjusted using the Benjamini–Hochberg procedure to control the false discovery rate.

For the predictor-matched H1 contrasts and the H2 comparisons, Diebold–Mariano results were complemented by a paired moving-block bootstrap of differences in MAE. Absolute-error differences were resampled in contiguous 12-month blocks using 2000 replicates, and empirical 95% confidence intervals were obtained from the bootstrap distribution. A paired difference was considered statistically supported by the bootstrap analysis when its 95% confidence interval did not include zero. Statistical tests were evaluated at *α* = 0.05, but interpretation also considered the magnitude and direction of the error differences rather than relying exclusively on statistical significance. Non-significant tests were interpreted as insufficient evidence of different predictive accuracy and not as evidence of model equivalence.

### Hydroclimatic structure and temporal change

The statistical properties of the monthly discharge series were examined to evaluate whether the post-2012 period differed from the preceding record. Descriptive statistics, including mean, median, standard deviation, variance, coefficient of variation, quartiles, minimum, and maximum, were calculated separately for January 1966–December 2011 and January 2012–April 2019 using observed discharge values.

Differences in the seasonal cycle were examined by calculating calendar-month means, medians, and standard deviations for the pre-2012 and post-2012 periods. Serial dependence was characterized using autocorrelation at lags 1 and 12. Its temporal stability was further examined through autocorrelation calculated within a 10-year moving window. Pairwise-complete observations were used when calculating window-specific autocorrelations.

Potential abrupt changes were examined using the Pettitt, Buishand *U*, and Standard Normal Homogeneity tests. Monotonic temporal change was evaluated using the Mann–Kendall test and Sen’s slope. Missing discharge observations were omitted test-wise, and no values were imputed for these analyses.

These complementary analyses were used to determine whether the post-2012 period showed changes in discharge magnitude, variability, seasonality, or temporal dependence. The period was not classified as formally non-stationary unless supported consistently by the statistical evidence.

### Software and reproducibility

All data processing, statistical analyses, model fitting, validation, and graphical representations were performed in R version 4.5.2 (R Core Team, 2025) under Windows 11. Data organization and manipulation used the dplyr, tidyr, purrr, readr, tibble, and lubridate packages. Figures were generated using ggplot2 and scales.

ARMA, ARIMA, SARIMA, seasonal-naive predictions, forecast-accuracy procedures, and Diebold–Mariano tests were implemented using the forecast package. MLP models were fitted using nnet. Stationarity and temporal-structure analyses used tseries, trend, urca, and zoo. Block-bootstrap procedures were implemented within the analysis script using base R and the tidyverse workflow.

All stochastic procedures used fixed random seeds; the principal seed was 123. Package versions, selected model specifications, fold-level cross-validation results, data-audit outputs, and the scripts used to generate all tables and figures are provided in the Supplementary Material and in the accompanying repository: https://github.com/Luisamsv/Serinha-mdata.

## Results and discussion

### Hydroclimatic context and temporal structure

Mean discharge decreased from 5.52 m^3^ s⁻^1^ during the pre-2012 period to 4.71 m^3^ s⁻^1^ between January 2012 and April 2019, corresponding to a reduction of approximately 14.8% (Table S1). Thirteen post-2012 months exhibited discharge below the 10th percentile of the pre-2012 distribution. Direct monthly comparisons indicated lower discharge after 2012: both the Welch mean comparison and the Wilcoxon location test remained significant after Benjamini–Hochberg adjustment (adjusted *p* = 0.020 for both tests; Table S9, Panel B). The timing of this reduction was consistent with the prolonged drought affecting Northeast Brazil, although the observational comparison does not establish drought as its exclusive cause (Marengo et al., 2017).

Despite the reduction in discharge magnitude, the Brown–Forsythe test did not detect a significant difference in variance between periods (adjusted *p* = 0.806), and the month-by-period interaction was not significant (adjusted *p* = 0.933; Table S9, Panel B). The monthly climatology consequently retained a broadly similar annual pattern, although discharge was lower during several post-2012 months and the corresponding estimates were more uncertain because of the shorter observational record (Figure S8). These results indicate a downward shift in discharge magnitude without detectable changes in variance or in the general shape of the monthly climatology.

The complete monthly series exhibited marked temporal persistence and a recurrent annual signal. The autocorrelation function (ACF), which summarizes correlation across time lags, showed significant dependence at short lags and at the 12-month lag, whereas the partial autocorrelation function (PACF), which isolates the association at each lag after accounting for shorter lags, primarily indicated short-term dependence (Figure S1). Short-term dependence remained evident in the period-specific estimates: the lag-1 ACF was 0.616 before 2012 and 0.734 after 2012. In contrast, the lag-12 ACF decreased from 0.168 to 0.100. These point estimates suggest that month-to-month persistence remained substantial while annual-lag dependence was weaker during the shorter post-2012 record; however, they do not demonstrate statistical stability or equivalence of the autocorrelation structure between periods. The temporal characteristics of the complete series supported the use of lagged-discharge predictors and motivated the evaluation of both non-seasonal and seasonal stochastic formulations.

Tests applied to annual discharge did not identify an unequivocal structural breakpoint (Table S9, Panel A). The Pettitt, Buishand *U*, and Standard Normal Homogeneity tests did not reach the conventional 5% significance threshold. Their estimated candidate change points occurred in 1981 or 1985 rather than in 2012 and therefore did not support an abrupt breakpoint associated specifically with the post-2012 period. The Mann–Kendall test indicated a negative but non-significant long-term trend (*Z* =  − 1.444, *p* = 0.149), while Sen’s slope was − 0.0232 m^3^ s⁻^1^ yr⁻^1^. The negative slope describes the estimated direction and magnitude of change but does not constitute statistical evidence of a monotonic long-term decline.

The significant reduction detected in the monthly comparisons alongside the absence of a significant annual breakpoint is not contradictory. The monthly tests evaluated differences in the overall discharge distribution using a substantially larger number of observations, whereas the structural-change tests assessed whether the annual series contained a discrete and persistent shift at an unknown point in time. Annual aggregation and the relatively short post-2012 segment also reduced the information available for detecting a breakpoint specifically associated with 2012. Thus, the results support a reduction in discharge magnitude during the post-2012 period but provide insufficient evidence to classify this change as an abrupt hydrological regime shift.

Taken together, the results demonstrate a significant reduction in monthly discharge magnitude after 2012 but do not establish an abrupt annual breakpoint, a significant monotonic trend, a change in variance, or disruption of the general monthly pattern. Short-term persistence remained evident in both periods, whereas the available results do not support the stronger conclusion that temporal dependence remained unchanged. The coexistence of reduced discharge magnitude and continued short-term persistence is consistent with evidence from other Brazilian streamflow records in which temporal dependence remained detectable despite multiyear drought and flood sequences (Araujo & Celeste, 2019). These findings support H3 with respect to discharge magnitude: the analyses detected a significant post-2012 reduction in discharge, but did not detect changes in variance or in the general seasonal pattern, while evidence for changes in temporal dependence remained inconclusive. Accordingly, T1 and T2 were treated as contrasting temporal calibration configurations rather than as samples separated by a formally demonstrated hydrological regime shift.

### Performance of ARMA, ARIMA, and SARIMA models

Among the stochastic candidates, cross-validation performance was similar under T1 but differed markedly under T2 (Table 1; Tables S3–S5). Under T1, the selected ARMA(1,0,2), ARIMA(1,1,3), and SARIMA(2,1,2)(2,0,0)[12] models yielded CV RMSE values of 1.957, 1.957, and 1.911 m^3^ s⁻^1^, respectively. Thus, SARIMA produced the lowest error, although its advantage over ARMA and ARIMA was small. Under T2, SARIMA(3,1,2)(1,0,1)[12] achieved a CV RMSE of 1.309 m^3^ s⁻^1^ and a CV MAE of 1.092 m^3^ s⁻^1^, representing approximately 30% lower RMSE and 14–15% lower MAE than the selected ARMA and ARIMA models. This pronounced difference identified the T2 SARIMA configuration as the strongest stochastic candidate during rolling-origin model selection.

**Table 1:** Best-performing model configurations selected by rolling-origin cross-validation under baseline (T1) and drought-focused (T2) calibration frameworks. Q1, Q2, Q3, and Q12 denote discharge at lags 1, 2, 3, and 12 months, respectively; P1 and P2 denote precipitation at lags 1 and 2 months. MLP architecture is expressed as input nodes–hidden neurons–output node. CV RMSE and CV MAE correspond to mean rolling-origin cross-validation errors. The number of successful folds is reported relative to the total number of eligible temporal folds

Model class	Period	Selected configuration	Predictors/architecture	CV RMSE (m^3^ s⁻^1^)	CV MAE (m^3^ s⁻^1^)	Successful folds
ARMA	T1	ARMA(1,0,2)	—	**1.9570**	**1.3685**	40/40
ARMA	T2	ARMA(1,0,2)	—	**1.8681**	**1.2742**	39/39
ARIMA	T1	ARIMA(1,1,3)	—	**1.9565**	**1.3291**	40/40
ARIMA	T2	ARIMA(2,1,4)	—	**1.8783**	**1.2864**	39/39
SARIMA	T1	SARIMA(2,1,2)(2,0,0)[12]	—	**1.9112**	**1.3152**	38/40
SARIMA	T2	SARIMA(3,1,2)(1,0,1)[12]	—	**1.3094**	**1.0918**	38/39
Linear regression	T1	M6	Q1 + Q2 + Q3 + P1 + P2	**1.9583**	**1.3573**	40/40
Linear regression	T2	M1	Q1 + P1	**1.9179**	**1.2667**	39/39
MLP univariate	T1	U3	Q1 + Q2 + Q3; 3–8–1, decay = 0	**1.7132**	**1.1609**	40/40
MLP univariate	T2	U5	Q1 + Q2 + Q3 + Q12; 4–4–1, decay = 0.01	**1.7256**	**1.2313**	37/37
MLP multivariate	T1	M3	Q1 + Q2 + P1 + P2; 4–4–1, decay = 0.01	**1.7942**	**1.2102**	40/40
MLP multivariate	T2	M4	Q1 + Q2 + Q12 + P1 + P2; 5–10–1, decay = 0.001	**1.7241**	**1.2831**	37/37

ARMA and ARIMA showed comparable cross-validation performance under both temporal configurations, indicating limited and inconsistent benefit from non-seasonal differencing (Table 1; Tables S3–S4). Under T1, ARIMA(1,1,3) and ARMA(1,0,2) produced nearly identical errors. Under T2, selection shifted to the higher-order ARIMA(2,1,4) formulation, but its performance remained similar to that of the simpler ARMA(1,0,2) model. Seasonal terms likewise did not provide uniform gains: their contribution was modest under T1 but substantial during rolling-origin model selection under T2. Together with the significant 12-month autocorrelation, this result indicates that explicit annual dependence was informative for the selected T2 configuration at the model-selection stage.

Increasing stochastic-model complexity therefore did not uniformly improve rolling-origin cross-validation performance. Higher non-seasonal orders provided little or no consistent advantage, supporting parsimonious order selection when additional parameters do not yield measurable predictive gains (Hyndman & Khandakar, 2008). The selected T2 SARIMA model represented an important configuration-specific exception. Previous streamflow applications have similarly shown that SARIMA performance can be sensitive to the length and composition of the training record, including susceptibility to overfitting when calibration data are limited (Alonso Brito et al., 2021). This sensitivity reinforces the importance of temporally ordered out-of-sample evaluation under potentially nonstationary conditions (Cerqueira et al., 2020). Whether the pronounced cross-validation advantage of the T2 SARIMA model generalized beyond the model-selection stage was therefore examined later through independent validation.

The pronounced cross-validation advantage of the selected T2 SARIMA model did not persist during independent one-step validation. SARIMA achieved an RMSE of 1.824 m^3^ s⁻^1^ and an MAE of 1.314 m^3^ s⁻^1^, values similar to those of ARMA and ARIMA (Fig. 2; Table S14). Thus, the selection-stage advantage of the seasonal formulation was attenuated outside the rolling-origin model-selection procedure, reinforcing the distinction between cross-validation ranking and independent predictive performance.

### Performance of the univariate multilayer perceptron model

Rolling-origin cross-validation showed that the performance of the univariate MLP depended on the temporal configuration and lag structure (Table 1; Table S6). Under T1, the selected U3 model, using Q1, Q2, and Q3 in a 3–8–1 architecture, achieved a CV RMSE of 1.713 m^3^ s⁻^1^ and a CV MAE of 1.161 m^3^ s⁻^1^, representing the lowest selection-stage errors among all model families. Under T2, the selected U5 model incorporated Q12 in addition to Q1, Q2, and Q3 and achieved a CV RMSE of 1.726 m^3^ s⁻^1^ and a CV MAE of 1.231 m^3^ s⁻^1^. The inclusion of Q12 in the selected T2 configuration indicates that annual discharge memory provided useful information during model selection. Nevertheless, the T2 univariate MLP did not match the substantially lower cross-validation errors of the selected SARIMA model, demonstrating that nonlinear structure did not confer a uniform advantage across temporal configurations.

Additional sensitivity analyses did not support the use of substantially longer continuous lag windows. Under T1, the best univariate MLP using continuous 12- and 24-month discharge lags produced CV RMSE values of 2.065 and 2.363 m^3^ s⁻^1^, respectively, compared with 1.713 m^3^ s⁻^1^ for the selected U3 model. Under T2, the corresponding values were 1.919 and 2.238 m^3^ s⁻^1^, compared with 1.726 m^3^ s⁻^1^ for U5. Thus, increasing the number of lagged inputs did not improve cross-validation performance and instead favored the more parsimonious lag structures selected in the primary analysis. These findings support the selected parsimonious lag structures, as extending the input window to 12 or 24 months did not improve predictive performance. However, potential gains from deeper feedforward or recurrent architectures cannot be excluded.

Importantly, the selection-stage ranking did not transfer uniformly to independent one-step predictions. Over common prediction dates under T1, the univariate MLP produced an RMSE of 1.708 m^3^ s⁻^1^ and an MAE of 1.292 m^3^ s⁻^1^, whereas the stochastic models produced RMSE values of 1.643–1.664 m^3^ s⁻^1^ and MAE values of 1.201–1.237 m^3^ s⁻^1^. Under T2, the numerical ordering was reversed: the univariate MLP achieved an RMSE of 1.753 m^3^ s⁻^1^ and an MAE of 1.267 m^3^ s⁻^1^, compared with RMSE values of 1.821–1.825 m^3^ s⁻^1^ and MAE values of 1.300–1.315 m^3^ s⁻^1^ for the stochastic models. Nevertheless, paired Diebold–Mariano tests detected no significant differences between the univariate MLP and ARMA, ARIMA, or SARIMA under either temporal configuration after Benjamini–Hochberg correction (adjusted *p* = 0.601–0.665; Table 2). The reversal in numerical ranking therefore indicates configuration-dependent relative performance but does not establish statistically superior predictive accuracy for either the univariate MLP or the stochastic models.

**Table 2:** Diebold–Mariano tests comparing one-step-ahead predictive accuracy between the selected stochastic models and the univariate MLP and between linear regression and the multivariate MLP under the T1 and T2 temporal configurations. Tests were conducted over common validation dates using absolute-error loss and a two-sided alternative. The loss differential was calculated as the loss of the first model minus that of the second model; therefore, negative DM statistics favor the first model and positive values favor the second. Raw p-values were adjusted across the eight comparisons using the Benjamini–Hochberg procedure. No comparison was statistically significant at adjusted *p* < 0.05

Period	Comparison	*N*	DM	Raw *p*-value	BH-adjusted *p*-value
T1	ARMA vs MLP univariate	152	−0.760	0.448	0.601
T1	ARIMA vs MLP univariate	152	−1.311	0.192	0.601
T1	SARIMA vs MLP univariate	152	−1.192	0.235	0.601
T1	Linear regression vs MLP multivariate	143	−1.299	0.196	0.601
T2	ARMA vs MLP univariate	84	0.804	0.424	0.601
T2	ARIMA vs MLP univariate	84	0.758	0.450	0.601
T2	SARIMA vs MLP univariate	84	0.553	0.582	0.665
T2	Linear regression vs MLP multivariate	78	−0.434	0.666	0.666

Exploratory sensitivity analyses further indicated that MLP performance depended on network specification and optimization. After hidden-layer size and weight decay were re-optimized within each activation, tanh minimized CV RMSE in three of four period-by-information-domain comparisons, whereas logistic performed best for T1 discharge-only; ReLU did not minimize CV RMSE but yielded the lowest CV MAE in both T2 domains. Because the custom logistic BFGS implementation did not fully reproduce the primary nnet predictions (*r* = 0.883–0.984; RMSE differences = 1.3–13.4%), these activation results were not used to reselect the primary models. In a separate custom backpropagation analysis, very low learning rates generally degraded performance, whereas rates from 0.005 to 0.1 produced broadly similar CV errors and no rate showed a uniform advantage over 0.01. Because most fits reached the 2000-epoch training limit, the learning-rate results should be interpreted as conditional on the adopted training budget.

The reversal between rolling-origin model selection and independent validation is more informative than a simple claim of MLP superiority. It indicates that the relative ranking of the nonlinear and stochastic models depended on the temporal configuration, evaluation stage, and validation sample. This context-dependent behavior is consistent with hydrological neural-network research showing that performance depends on input selection, network configuration, training procedure, and validation design rather than on nonlinear architecture alone (Maier et al., 2010; Zounemat-Kermani et al., 2020). The absence of statistically significant differences should not be interpreted as model equivalence, but it provides insufficient evidence to conclude that the univariate MLP was consistently more accurate than the stochastic alternatives.

### Univariate versus multivariate MLP configurations

The monthly climatological cycles showed that precipitation and discharge shared a seasonal structure, although their maxima did not coincide exactly. Precipitation was relatively higher from March to July, whereas mean discharge reached its maximum in July, consistent with an aggregated and potentially delayed hydrological response at the monthly scale (Figure S9). Supplementary analyses indicated a statistically significant but moderate association between precipitation and discharge. Spearman’s correlations between monthly discharge and precipitation were moderate at lag 0 (*ρ* = 0.434, *N* = 628) and lag 1 (*ρ* = 0.401, *N* = 626), decreasing to *ρ* = 0.296, 0.280, and 0.146 at lags 2, 3, and 12, respectively (Table S10). The contemporaneous precipitation–discharge relationship is illustrated in Figure S3A. All correlations remained statistically significant after Benjamini–Hochberg correction. The cross-correlation analysis produced its largest coefficients at lag 0 (CCF = 0.468) and lag − 1 (CCF = 0.414), the latter corresponding to precipitation preceding discharge by one month (Figure S3B). Thus, precipitation contained information relevant to monthly discharge variability, but part of this information may already have been indirectly represented by antecedent discharge. Statistical association between precipitation and discharge therefore did not necessarily translate into incremental predictive skill when precipitation was included among the MLP inputs.

Rolling-origin cross-validation provided no consistent evidence that the selected multivariate MLP configuration outperformed the selected univariate configuration (Table 1; Tables S6–S7). Under T1, the univariate MLP achieved lower CV RMSE and MAE than the multivariate configuration (1.713 versus 1.794 m^3^ s⁻^1^ and 1.161 versus 1.210 m^3^ s⁻^1^, respectively). Under T2, the multivariate MLP produced only a negligible reduction in CV RMSE relative to the univariate model (1.724 versus 1.726 m^3^ s⁻^1^), whereas its CV MAE was higher (1.283 versus 1.231 m^3^ s⁻^1^). Consequently, the relative performance of the two configurations depended on the error metric and did not indicate a uniform advantage for the selected multivariate model.

**Fig. 3:**
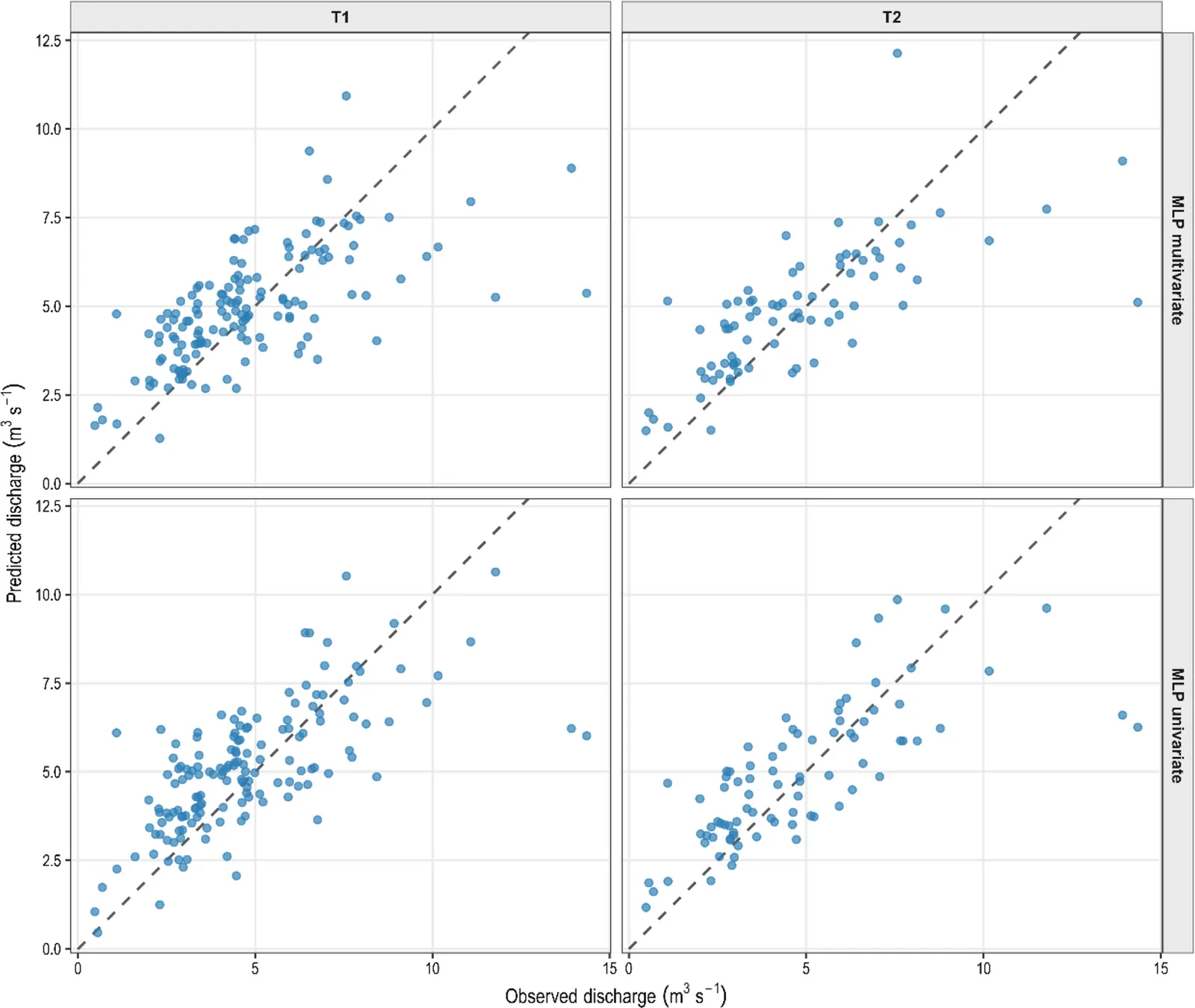
Observed versus predicted monthly river discharge for the selected multilayer perceptron (MLP) models under the baseline (T1) and drought-focused (T2) hydroclimatic configurations. Columns represent the temporal configuration (T1 and T2), and rows represent the selected MLP type (multivariate and univariate). The dashed line indicates the 1:1 relationship between observed and predicted values

The same pattern emerged from independent validation restricted to dates for which predictions from both MLP configurations were available (Table S11). Under T1, the univariate and multivariate models produced RMSE values of 1.764 and 1.801 m^3^ s⁻^1^, respectively, across 143 common observations, while their MAE values were nearly identical (1.306 and 1.306 m^3^ s⁻^1^). Under T2, the univariate MLP had a lower RMSE than the multivariate model (1.838 versus 1.891 m^3^ s⁻^1^), whereas the multivariate model had a slightly lower MAE (1.289 versus 1.300 m^3^ s⁻^1^) across 77 common observations. These small and metric-dependent differences, together with the observed-versus-predicted relationships shown in Fig. 3, provided no consistent evidence of a systematic predictive advantage for the selected configuration containing antecedent precipitation.

Nevertheless, because the selected univariate and multivariate configurations also differed in network architecture and discharge-lag composition, their comparison does not isolate the effect of precipitation alone. Instead, it describes the performance of the complete configurations selected independently through rolling-origin cross-validation. The limited incremental value observed for the selected multivariate configuration does not imply that precipitation was hydrologically irrelevant. Rather, its predictive contribution may have been constrained by the monthly temporal resolution, the moderate lagged association with discharge, and redundancy between antecedent precipitation and information already contained in discharge persistence. Previous hydrological neural-network studies have similarly shown that the contribution of exogenous climatic predictors depends on temporal aggregation, input selection, redundancy among predictors, network specification, and validation design (Adamowski & Karapataki, 2010; Maier et al., 2010; Mehr et al., 2015; Zounemat-Kermani et al., 2020).

Both pseudo-out-of-sample calibration predictions and independent validation results revealed a systematic compression of the predicted discharge range. Across T1 and T2, both MLP configurations consistently overestimated low flows and underestimated high-flow events, with the largest RMSE values generally occurring in the high-flow class (Table S12). During pseudo-out-of-sample calibration, mean bias ranged from +1.31 to +1.36 m^3^ s⁻^1^ for low-flow predictions and from −1.26 to −1.71 m^3^ s⁻^1^ for high-flow predictions. The same directional pattern persisted during independent validation, with low-flow bias ranging from +0.96 to +1.22 m^3^ s⁻^1^ and high-flow bias from −0.86 to −1.23 m^3^ s⁻^1^ (Table S12; Figure S4). This consistency indicates that compression of the predicted discharge range was already present in temporally withheld predictions within the calibration periods and was therefore not a pattern arising exclusively during independent validation. The inclusion of antecedent precipitation did not eliminate this limitation, indicating that the selected multivariate predictor sets did not resolve the principal difficulty of the MLP models in representing discharge extremes.

### Performance and role of the linear regression model

During rolling-origin model selection, the linear regression candidates produced errors of the same general order of magnitude as the other model families, although they were not the highest-ranked models under either temporal configuration (Table 1; Table S8). Under T1, the selected M6 model, based on Q1, Q2, Q3, P1, and P2, achieved a CV RMSE of 1.958 m^3^ s⁻^1^ and a CV MAE of 1.357 m^3^ s⁻^1^. Under T2, selection shifted to the more parsimonious M1 formulation containing Q1 and P1, which achieved a CV RMSE of 1.918 m^3^ s⁻^1^ and a CV MAE of 1.267 m^3^ s⁻^1^. The change in selected predictor structure indicates that a larger set of antecedent variables was not consistently favored across the two calibration configurations.

**Fig. 4:**
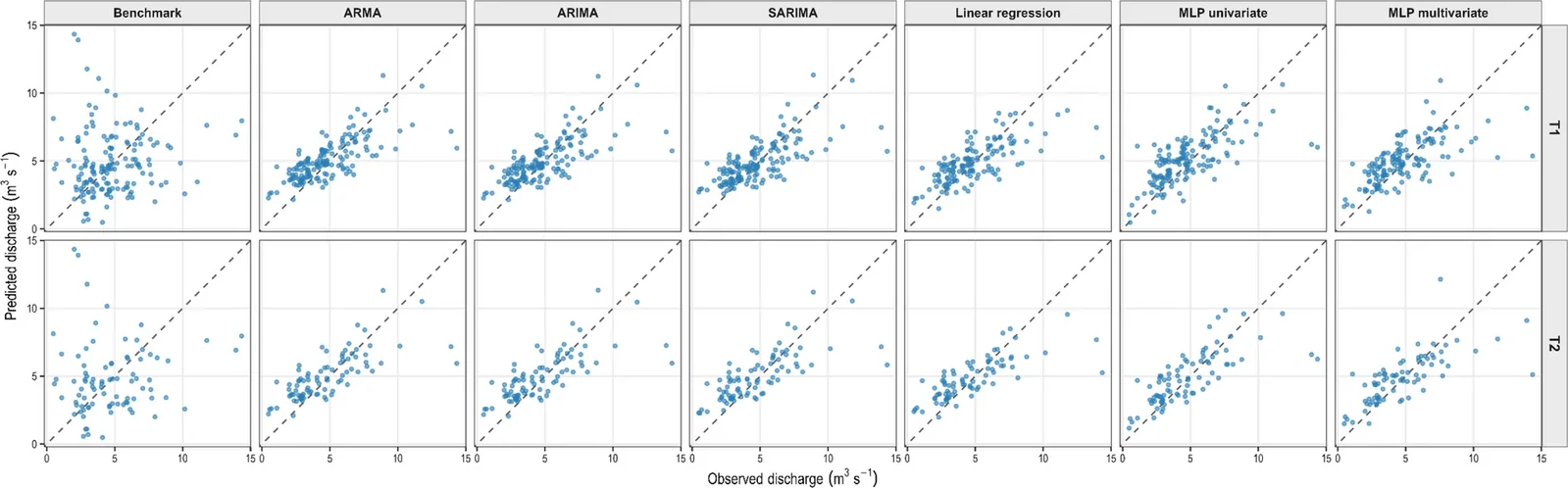
Observed versus predicted monthly river discharge for the seasonal-naive benchmark, stochastic time-series models (ARMA, ARIMA, and SARIMA), linear regression, and multilayer perceptron (MLP) models during independent one-step validation. T1 was evaluated from January 2006 to April 2019, whereas T2 was evaluated from January 2012 to April 2019. Columns represent the temporal configurations, and rows represent the model families. The dashed line indicates the 1:1 relationship between observed and predicted discharge. Deviations from this line indicate under- or overprediction, with larger departures generally occurring at higher observed flows. Only months with finite observed and predicted values are shown

In independent one-step validation, linear regression remained competitive rather than functioning as a lower-bound benchmark (Table S14). Under T1, it achieved an RMSE of 1.693 m^3^ s⁻^1^ and an MAE of 1.235 m^3^ s⁻^1^ across 143 valid predictions. Its RMSE was slightly higher than those of the selected stochastic models, whereas its MAE was of a similar magnitude. Both errors were lower than those of the selected MLP configurations. Under T2, linear regression achieved an RMSE of 1.811 m^3^ s⁻^1^ and an MAE of 1.269 m^3^ s⁻^1^ across 82 valid predictions. Its RMSE was only slightly higher than that of the univariate MLP and lower than those of the remaining selected models, while its MAE was nearly identical to that of the univariate MLP and lower than those of the stochastic and multivariate MLP models. Thus, the relative ranking obtained during model selection did not transfer uniformly to independent validation. Because T1 and T2 involved different validation periods and numbers of available predictions, however, these values describe performance within each configuration and should not be interpreted as a direct test of temporal robustness. Across the selected model families, observed-versus-predicted relationships also showed larger departures from the 1:1 line at higher observed discharge (Fig. 4), consistent with the flow-dependent error patterns identified above.

To distinguish predictor availability from model structure, a complementary analysis compared linear regression and MLP models using identical discharge-only and discharge-plus-precipitation inputs and the same validation dates (Table S13). Under T1, for the discharge-plus-precipitation predictor set, linear regression achieved significantly lower errors than the corresponding MLP model (RMSE: 1.693 versus 1.948 m^3^ s⁻^1^; MAE: 1.235 versus 1.456 m^3^ s⁻^1^). The difference in MAE, calculated as MLP minus linear regression, was 0.221 m^3^ s⁻^1^, with a 95% moving-block bootstrap confidence interval of 0.098–0.344 m^3^ s⁻^1^, and the Benjamini–Hochberg-adjusted Diebold–Mariano p-value was 0.0027 (Table S13). This was the only predictor-matched comparison showing a statistically significant difference.

For the remaining comparisons, linear regression produced lower RMSE values, but differences in predictive accuracy were not statistically significant after adjustment for multiple testing (Table S13). Under T1 with discharge-only predictors, the linear and MLP models achieved RMSE values of 1.653 and 1.730 m^3^ s⁻^1^, respectively, with an adjusted p-value of 0.881. Under T2, their performance was similar for the discharge-plus-precipitation inputs (RMSE: 1.847 versus 1.851 m^3^ s⁻^1^; MAE: 1.256 versus 1.276 m^3^ s⁻^1^). For discharge-only inputs under T2, linear regression had a lower RMSE, whereas the MLP had a marginally lower MAE, further demonstrating that the comparison depended on the error metric considered. The corresponding confidence intervals for the MAE differences included zero in all three cases.

Taken together, the predictor-matched results provided no general evidence that nonlinear transformation alone improved out-of-sample predictive accuracy. Indeed, under the T1 discharge-plus-precipitation configuration, the simpler linear formulation performed significantly better. This result does not imply that discharge generation in the basin is inherently linear or that nonlinear hydrological processes are absent. Rather, the observed pattern may reflect constraints associated with the monthly temporal resolution, available sample size and predictor sets, and potential redundancy between antecedent precipitation and discharge, under which the additional flexibility of the MLP did not consistently translate into better independent predictions. Neural-network performance in hydrological applications depends strongly on input selection, network specification, calibration strategy, and validation design (Maier et al., 2010; Zounemat-Kermani et al., 2020), while increasing model complexity does not necessarily improve out-of-sample performance and may increase susceptibility to overfitting (Orth et al., 2015).

Linear regression therefore served as an interpretable reference model, but not simply as a lower bound on predictive performance. Its transparent structure provided a baseline for separating the contribution of predictor information from model flexibility, while its competitive validation performance demonstrated that increased complexity did not automatically improve generalization. Comparative streamflow assessments should consider predictive accuracy together with interpretability, error structure, uncertainty, and validation strategy (Shortridge et al., 2016). More broadly, the absence of a universally superior modeling approach reinforces the need for rigorous, problem-specific benchmarking rather than assuming an intrinsic advantage of nonlinear architectures (Papacharalampous & Tyralis, 2022). Accordingly, the predictor-matched validation results did not support H1’s expectation of a general predictive advantage of nonlinear models over linear formulations. As an overall reference, the seasonal-naive benchmark showed substantially larger one-step validation errors than the fitted model families, with RMSE values of 3.145 m^3^ s⁻^1^ under T1 and 3.524 m^3^ s⁻^1^ under T2 (Table S14). Thus, the fitted models captured predictive structure beyond simple repetition of discharge from the same month of the previous year.

### Generalization, temporal robustness, and uncertainty of MLP configurations

**Fig. 5:**
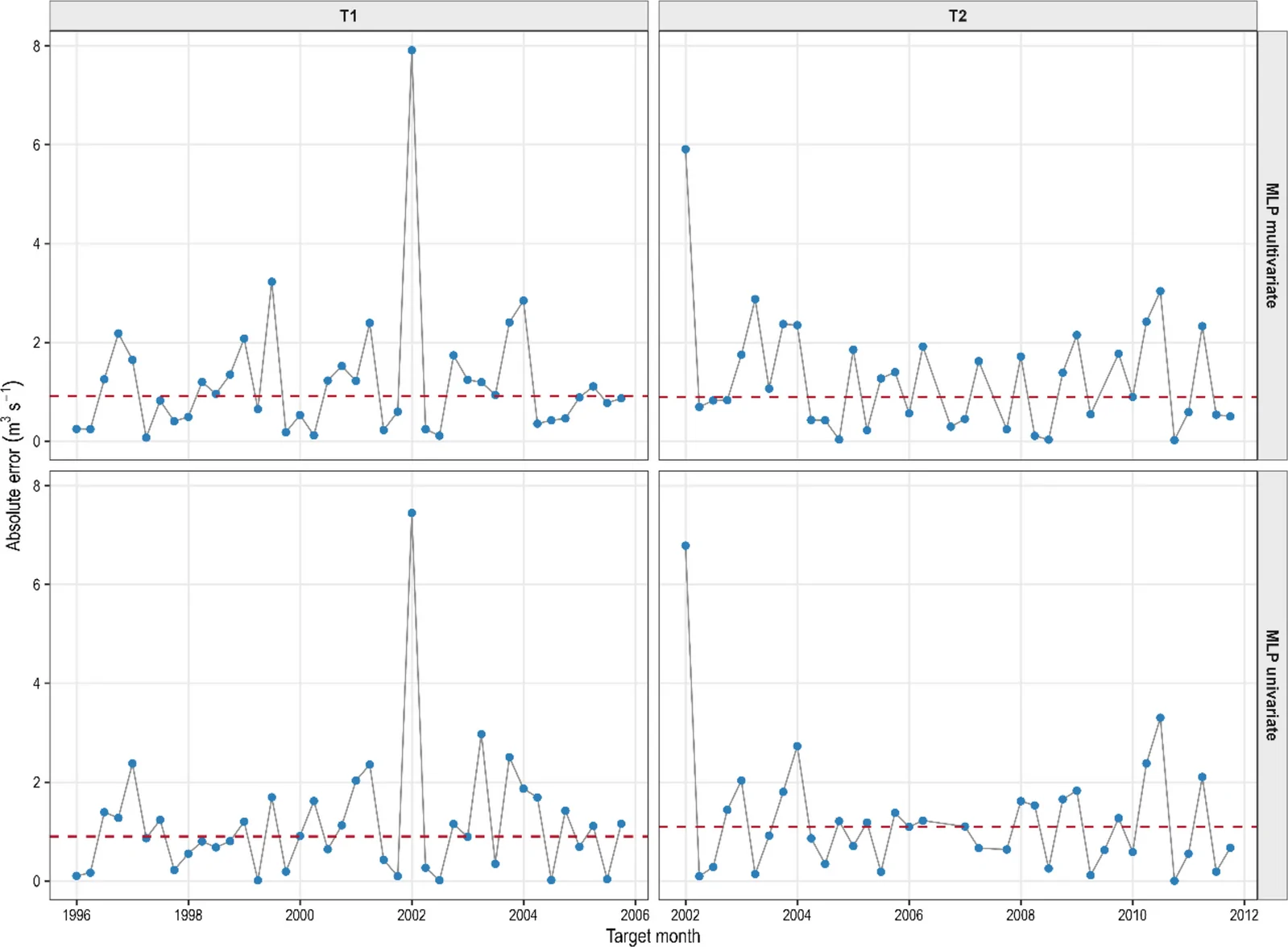
Rolling-origin cross-validation errors for the selected multilayer perceptron (MLP) models under the baseline (T1) and drought-focused (T2) configurations. Points represent the absolute prediction error for each target month, with panels corresponding to the multivariate and univariate MLP configurations. The red dashed line indicates the median absolute error within each model and temporal configuration. Isolated error peaks identify individual poorly predicted months, whereas visual inspection revealed no evident monotonic increase in errors across successive folds

**Fig. 6:**
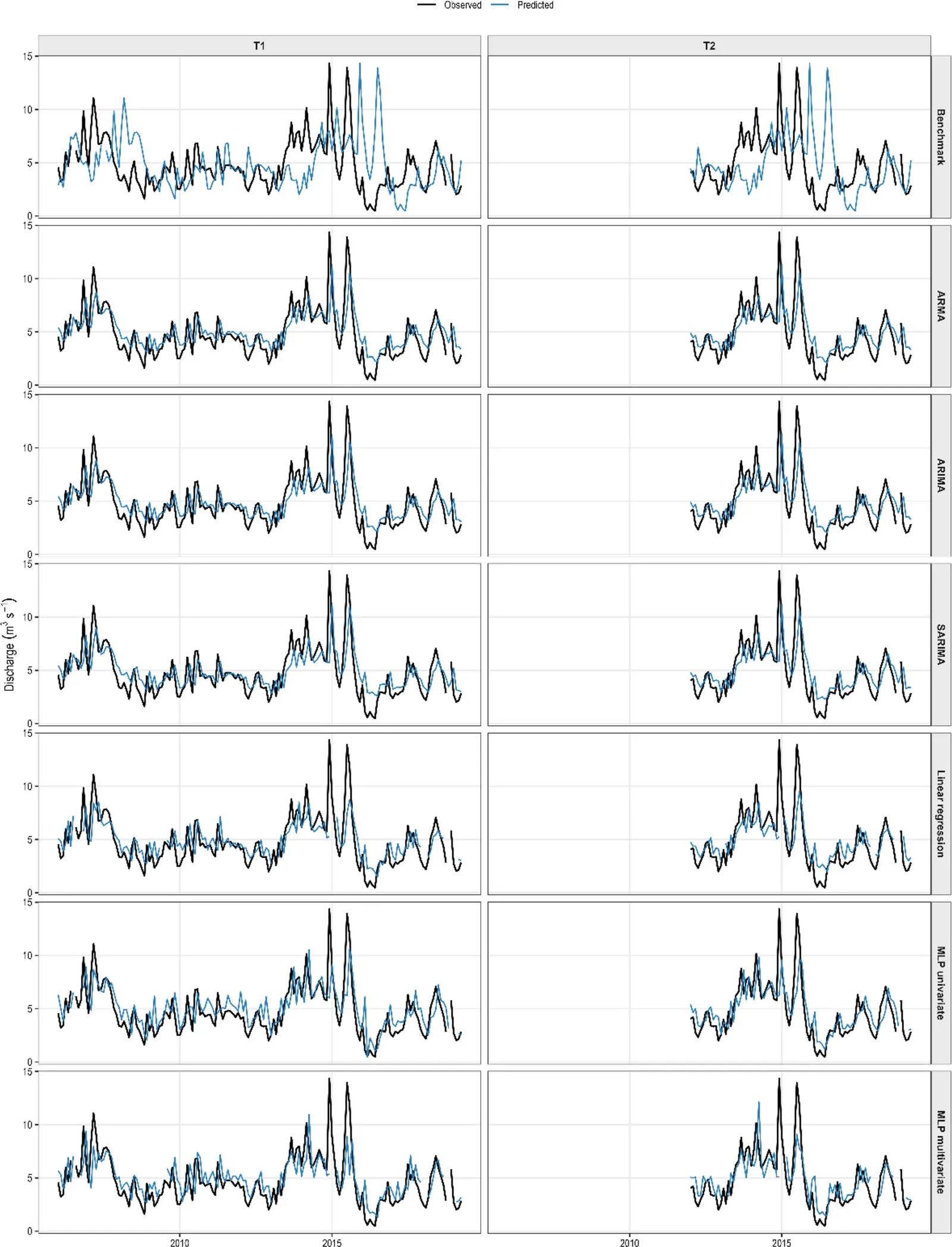
Observed and one-step-ahead predicted monthly river discharge for the seasonal-naive benchmark, stochastic time-series models (ARMA, ARIMA, and SARIMA), linear regression, and multilayer perceptron (MLP) models during independent validation. Columns show the complete evaluation periods for T1 (January 2006–April 2019) and T2 (January 2012–April 2019), whereas rows represent the model families. Black lines indicate observed discharge, and blue lines indicate model predictions. Only months with finite observed and predicted values are shown

The individual rolling-origin results showed temporal variation in MLP prediction errors, including isolated error peaks, but no evident monotonic increase across successive folds (Fig. 5). For the T1 univariate and multivariate MLP configurations, CV RMSE values of 1.713 and 1.794 m^3^ s⁻^1^ were close to the corresponding independent one-step validation values of 1.730 and 1.790 m^3^ s⁻^1^, respectively (Table 1; Table S14). Under T2, validation RMSE increased modestly relative to cross-validation for the univariate MLP (1.726 to 1.788 m^3^ s⁻^1^) and more noticeably for the multivariate MLP (1.724 to 1.884 m^3^ s⁻^1^), although changes in MAE were small. These results indicate reasonable transfer from rolling-origin model selection to independent one-step validation, but they do not demonstrate uniform temporal robustness across models or temporal configurations. The corresponding observed and one-step predicted monthly series for all model families are shown in Fig. 6. This distinction reinforces the importance of temporally ordered resampling and independent validation when evaluating time-series prediction models (Bergmeir et al., 2018; Cerqueira et al., 2020).

A comparison restricted to common prediction dates within the post-2012 validation period provided a more direct assessment of the T1 and T2 configurations under the same observed conditions (Figure S5). For the univariate MLP, RMSE decreased from 1.922 m^3^ s⁻^1^ under T1 to 1.788 m^3^ s⁻^1^ under T2, while MAE decreased from 1.346 to 1.268 m^3^ s⁻^1^. Differences were smaller for the multivariate MLP, with RMSE values of 1.895 and 1.884 m^3^ s⁻^1^ and MAE values of 1.252 and 1.287 m^3^ s⁻^1^ under T1 and T2, respectively. The common-date comparisons across the remaining model families likewise showed no uniform reduction in absolute error under T2: RMSE decreased only slightly for ARMA, ARIMA, SARIMA, and linear regression, whereas MAE increased for ARIMA and SARIMA and decreased only marginally for ARMA and linear regression. Scale-normalized metrics led to the same general conclusion. Although RSR was slightly lower under T2 for the fitted models, MASE increased for ARMA, ARIMA, SARIMA, and the multivariate MLP and decreased only for the univariate MLP among the model families with directly comparable post-2012 samples. Relative variance error also changed inconsistently: it moved closer to zero for ARMA (−0.600 to −0.593), ARIMA (−0.596 to −0.584), SARIMA (−0.600 to −0.586), and the multivariate MLP (−0.579 to −0.556), but farther from zero for the univariate MLP (−0.452 to −0.497). Thus, extending calibration through 2011 did not produce a uniform improvement across model families or performance criteria. Because the selected model specifications and calibration records also differed between T1 and T2, these comparisons do not isolate the individual effects of calibration length or calibration recency and should therefore not be interpreted causally.

Paired inference over the common post-2012 validation dates did not identify significant differences between the T1 and T2 configurations for either MLP after Benjamini–Hochberg correction. For the univariate MLP, the T2-minus-T1 difference in MAE was −0.078 m^3^ s⁻^1^ (95% moving-block bootstrap CI: −0.161 to 0.038 m^3^ s⁻^1^; DM =  −1.559; adjusted *p* = 0.363). For the multivariate MLP, the corresponding difference was 0.035 m^3^ s⁻^1^ (95% CI: −0.127 to 0.171 m^3^ s⁻^1^; DM = 0.552; adjusted *p* = 0.835). Among the remaining model families, only ARMA showed a statistically significant but numerically small reduction in MAE under T2 (ΔMAE =  −0.015 m^3^ s⁻^1^; DM =  −2.757; adjusted *p* = 0.043), with the bootstrap confidence interval only marginally excluding zero. No significant differences were detected for ARIMA, SARIMA, or linear regression. Accordingly, H2 was not supported as a general expectation of improved predictive performance under the updated T2 calibration configuration; instead, the inferential results indicated a limited and model-specific difference.

The relatively stable one-step performance did not extend uniformly to recursive prediction (Table S14). One-step predictions used observed antecedent discharge as an input, whereas recursive predictions reused previous model predictions whenever observed antecedent discharge was unavailable. Recursive errors were generally larger, and several configurations produced negative NSE values, indicating error accumulation when previous predictions were returned as inputs. For example, the univariate MLP produced recursive RMSE values of 3.810 and 3.401 m^3^ s⁻^1^ and NSE values of −1.688 and −0.648 under T1 and T2, respectively. Results for the multivariate MLP should be interpreted particularly cautiously because only 40 valid recursive predictions were available under T1 and 13 under T2.

The multihorizon analysis likewise showed a general increase in prediction errors and a decline in predictive skill as the distance from the last observed discharge value expanded across the evaluated 1-, 3-, 6-, 12-, and 24-month horizons (Figure S7). The changes were not monotonic for every model family, particularly at the longest horizon, for which the number of valid predictions was smaller and differed among models. Consequently, evidence of satisfactory one-step generalization should not be extrapolated to long recursive sequences. This behavior is consistent with the sensitivity of hydrological neural networks to prediction strategy, model configuration, and validation design (Maier et al., 2010; Zounemat-Kermani et al., 2020).

**Fig. 7:**
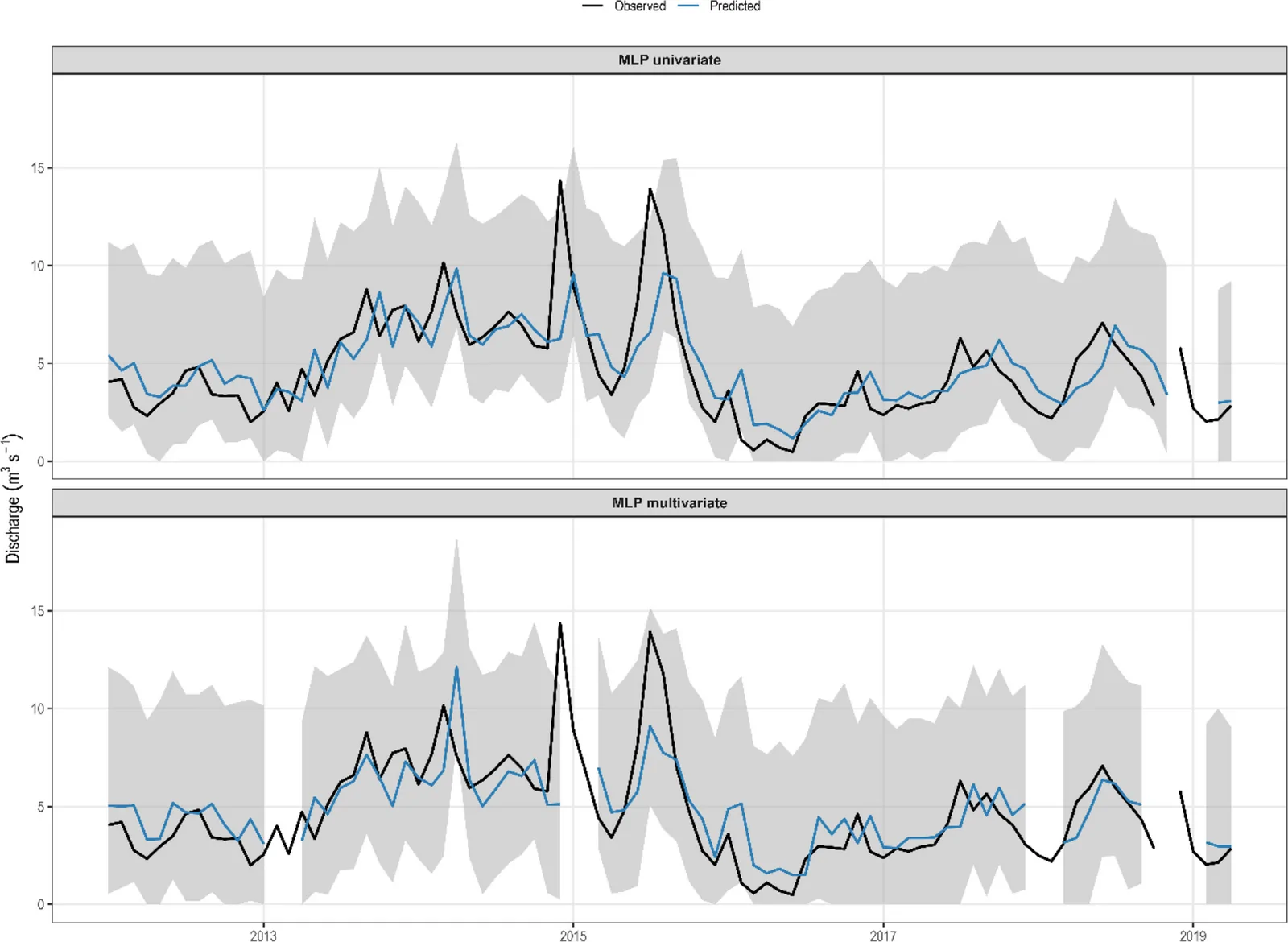
Observed and ensemble-mean one-step predicted monthly discharge for the selected univariate and multivariate multilayer perceptron (MLP) models under the T2 temporal configuration. Black lines indicate observed discharge, blue lines indicate the ensemble-mean predictions obtained across the 20 final MLP fits, and grey shaded areas represent the 95% residual-based predictive intervals generated using 2000 moving-block bootstrap realizations. Gaps indicate missing observations or unavailable predictor combinations. The intervals are conditional on the selected model configurations and hyperparameters

The 95% residual-based predictive intervals for one-step discharge predictions were generated using a 12-month moving-block bootstrap with 2000 replicates applied to pseudo-out-of-sample residuals obtained exclusively within the calibration periods. The intervals achieved empirical coverage close to or slightly above the nominal level (Fig. 7; Figure S6). Coverage was 97.4% and 97.2% for the T1 univariate and multivariate MLPs and 96.4% and 96.2% for the corresponding T2 models. Mean interval widths ranged from 9.18 to 10.18 m^3^ s⁻^1^, indicating slightly conservative but broadly stable conditional coverage across configurations. These intervals quantify residual-based predictive uncertainty conditional on the selected models and hyperparameters; they do not incorporate uncertainty arising from model selection, alternative architectures, or hyperparameter tuning. This distinction is important because bootstrap-based uncertainty estimates remain conditional on the data structure and modeling procedure from which they are generated (Efron & Tibshirani, 1998; Koutsoyiannis & Montanari, 2007).

Overall, the results support reasonable generalization of the selected MLP configurations for independent one-step prediction, while providing more limited evidence of robustness for recursive and longer-horizon prediction. Predictive robustness was therefore dependent on the prediction strategy, model configuration, and performance metric and should not be interpreted as an invariant property of the MLP framework. More broadly, the results reinforce the need to evaluate predictive accuracy together with temporal validation design, error propagation, and uncertainty rather than inferring robustness from a single evaluation strategy (Shortridge et al., 2016).

### Ecological implications for estuarine circulation and biogeochemical processes

The estuarine implications discussed here should be interpreted as conceptual inferences grounded in established estuarine theory and comparable empirical studies, rather than as site-specific predictions. The present study reconstructed river discharge at the Ituberá gauge but did not simulate estuarine circulation, salinity, residence time, sediment transport, or biogeochemical transformations. Consequently, the results identify changes in an important estuarine boundary condition but do not quantify the corresponding response of the Serinhaém estuary.

The discharge record provides evidence of lower river discharge at the Ituberá gauge during the post-2012 period. Mean monthly discharge from 2012 to April 2019 was approximately 15% lower than during the pre-2012 reference period, and 13 months exhibited discharge below the 10th percentile of the pre-2012 distribution (Table S1). The sample variance was numerically lower after 2012, although the Brown–Forsythe test did not detect a statistically significant difference between periods. However, the structural-change and trend tests did not provide statistically significant evidence of an abrupt breakpoint or monotonic long-term trend (Table S9). The post-2012 period should therefore be interpreted as an interval of comparatively reduced discharge rather than as a formally demonstrated hydrological regime shift. Moreover, because no model family was uniformly superior across model-selection and independent-validation stages, the ecological interpretation is based on the observed change in discharge conditions rather than on an assumed advantage of nonlinear reconstruction.

River discharge is a major boundary condition controlling estuarine circulation, longitudinal salinity gradients, stratification, residence time, and the transport of dissolved and particulate material (Dyer, 1997; Geyer & MacCready, 2014; Monismith et al., 2002). Persistent reductions in freshwater inflow may favor landward salinity penetration, modify vertical mixing and stratification, and alter the time available for sedimentation and biogeochemical processing. Nevertheless, the direction and magnitude of these responses depend on estuarine geometry, bathymetry, tidal forcing, ocean boundary conditions, wind, and the temporal distribution of river flow (Geyer & MacCready, 2014; Mclusky & Wolanski, 2012; Uncles et al., 2002). The approximately 15% reduction observed in the discharge record therefore cannot be translated directly into a specific displacement of the salinity limit or a proportional change in residence time without a calibrated hydrodynamic model.

Changes in freshwater inflow may also influence sediment retention, organic-matter processing, nutrient transformation, oxygen conditions, and the transport of particle-associated substances. Longer residence times and altered salinity and mixing conditions can modify the balance among transport, deposition, remineralization, and export in estuarine environments (Alongi, 2008; Mclusky & Wolanski, 2012). These processes are potentially relevant to the Serinhaém estuary, but they were not measured in the present study. They should therefore be regarded as testable implications for future hydrodynamic and biogeochemical investigations rather than as demonstrated ecological consequences of the post-2012 discharge reduction.

An additional spatial limitation is that the Ituberá gauge is located approximately 21.2 km upstream from the estuary mouth. Discharge measured or reconstructed at this station is therefore not necessarily identical to the total freshwater inflow reaching the estuary because intervening tributary contributions, water abstraction, groundwater exchange, evaporation, and channel losses were not quantified. The reconstructed series should consequently be interpreted as an upstream freshwater boundary indicator rather than as a complete estuarine water budget.

Despite these limitations, the reconstructed discharge series and its predictive uncertainty provide useful boundary information for subsequent estuarine applications. Future work could incorporate these discharge estimates into calibrated hydrodynamic and water quality models to test their consequences for salinity intrusion, stratification, residence time, sediment transport, and biogeochemical processing. Uncertainty in the discharge reconstruction should be propagated through such simulations rather than relying exclusively on a single deterministic inflow series. This approach would allow the conceptual implications identified here to be evaluated quantitatively for the Serinhaém estuary.

### Study limitations

This study reconstructs freshwater discharge at monthly resolution, which is suitable for assessing seasonal and interannual variability but does not resolve event-scale runoff, short-duration floods, tidal modulation, or other sub-monthly processes. Moreover, the analysis relied on a single discharge station located approximately 21.2 km upstream from the estuary mouth and a single precipitation gauge. The reconstructed discharge therefore does not necessarily represent total freshwater inflow to the estuary, because ungauged tributaries, downstream runoff, water withdrawals, and spatial rainfall variability were not quantified.

T1 and T2 differed simultaneously in calibration length, calibration recency, selected model configuration, and validation period. Comparisons restricted to common post-2012 dates standardized the evaluation interval but did not isolate the individual effects of calibration length, recency, or drought conditions. The relatively short post-2012 record also limited the information available for performance estimation and structural-change detection; consequently, failure to detect a significant breakpoint should not be interpreted as evidence of stationarity. In addition, the primary evaluation concerned one-step-ahead predictions conditioned on observed antecedent inputs. Recursive and longer-horizon errors were generally larger, and differences in missing observations and predictor availability resulted in unequal numbers of valid predictions among models.

The machine learning analysis was restricted to relatively simple, single-hidden-layer MLP architectures to maintain comparability and limit parameterization relative to the available sample size. Other network architectures and optimization procedures may yield different results. The exploratory activation- and learning-rate analyses further showed that neural-network performance can depend on implementation and optimization choices; because these analyses used custom implementations that were not fully equivalent to the primary *nnet* framework, they were treated as sensitivity checks and did not alter primary model selection. Likewise, non-significant Diebold–Mariano tests indicate insufficient evidence to reject equal predictive accuracy but do not demonstrate model equivalence. The residual-based predictive intervals were conditional on the selected models, hyperparameters, calibration residuals, block length, and bootstrap procedure and did not incorporate uncertainty associated with model selection, alternative predictors, or structural model choice.

Finally, the estuarine implications discussed here represent physically grounded inferences rather than direct hydrodynamic or biogeochemical predictions. Quantitative changes in salinity intrusion, residence time, stratification, sediment trapping, and material transport require explicit estuarine simulations supported by bathymetric, tidal, meteorological, salinity, and water quality data. The reconstructed monthly discharge series can provide an important upstream boundary condition for such analyses but should not be interpreted as a substitute for process-based estuarine modeling.

## Conclusions

This study compared linear regression, stochastic time-series models (ARMA, ARIMA, and SARIMA), and multilayer perceptron neural networks for reconstructing monthly river discharge at the Ituberá gauging station under contrasting temporal and hydroclimatic conditions. No model family was uniformly superior across rolling-origin model selection and independent validation. MLP configurations were frequently competitive and achieved low cross-validation errors, but their relative advantage depended on the temporal configuration, predictor set, and evaluation stage. In predictor-matched comparisons, nonlinear transformation did not consistently improve out-of-sample predictive accuracy, and one T1 discharge-plus-precipitation comparison significantly favored linear regression. H1 was therefore not supported as a general expectation of superior MLP performance.

H2 was not supported as a general pattern. Restricting the analysis to common post-2012 prediction dates showed that extending calibration through 2011 did not produce a uniform reduction in RMSE or MAE across model families, and the same conclusion was obtained from scale-normalized metrics and relative variance error. Although ARMA showed a statistically significant but numerically small reduction in MAE under T2, no consistent improvement was observed across the remaining model families. Thus, the longer and more recent T2 calibration record did not generally improve predictive performance under the post-2012 conditions.

The hydroclimatic analyses supported H3 with respect to discharge magnitude. Mean monthly discharge was approximately 15% lower after 2012, whereas no significant change in variance or in the general seasonal pattern was detected. Evidence for changes in temporal dependence remained inconclusive, and formal change-point and trend analyses did not establish an abrupt hydrological regime shift associated with 2012. The post-2012 period should therefore be interpreted as a hydroclimatically contrasting interval characterized primarily by reduced discharge magnitude rather than as a formally demonstrated alternative hydrological regime.

Selected models nevertheless provided useful one-step reconstructions of withheld monthly discharge, whereas recursive and longer-horizon performance was less robust because of error propagation and decreasing availability of valid predictor histories. Residual-based predictive intervals provided an additional measure of uncertainty around the selected MLP reconstructions, but these intervals remain conditional on the adopted models, predictors, and calibration procedure.

From an estuarine perspective, the reconstructed discharge series should be interpreted as an upstream freshwater boundary indicator rather than as a direct estimate of total freshwater inflow at the estuary mouth. The observed reduction in discharge may have implications for salinity intrusion, residence time, stratification, sediment transport, and biogeochemical processing, but these responses were not quantified in the present study and require explicit hydrodynamic and biogeochemical modeling. More broadly, the results show that monthly discharge reconstruction in data-scarce or discontinued-gauge tropical coastal basins benefits from rigorous temporal validation, predictor-matched benchmarking, and explicit characterization of predictive uncertainty rather than from assuming an intrinsic advantage of more complex nonlinear models.

## Supplementary Information

Below is the link to the electronic supplementary material.

ESM1 (DOCX 1.33 MB)

## Data Availability

No datasets were generated or analysed during the current study.

## Declarations

### Use of generative AI and AI-assisted technologies

During the preparation of this work, the authors used ChatGPT, developed by OpenAI, to assist with English-language revision and R code support. The authors critically reviewed and verified all AI-assisted content and take full responsibility for the final manuscript.
