# Supplementary material for: Modeling freshwater inflow to a tropical estuary using stochastic and neural-network approaches under contrasting hydroclimatic conditions in Brazil
Source: Environ Monit Assess. 2026 Oct 17;198(11):1211. doi: 10.1007/s10661-026-16034-1 (PMC13676660; doi:10.1007/s10661-026-16034-1)
Supplement: Supplementary file 1 [file 10661_2026_16034_MOESM1_ESM.docx]

**Modeling freshwater inflow to a tropical estuary using stochastic and neural-network approaches under contrasting hydroclimatic conditions in Brazil**

Luísa Maria de Souza Viana^a^, Eloá Côrrea Lessa Tostes^c^, Wendel Dias Constantino^a^, Felipe Henrique Rossi Luze^a^, Marcos Sarmet Moreira de Barros Salomão^a^, Taíse Bonfim de Jesus^b^, Carlos Eduardo Veiga de Carvalho^a^

^a^Programa de Pós-Graduação em Ecologia e Recursos Naturais, Laboratório de Ciências Ambientais, Centro de Biociências e Biotecnologia, Universidade Estadual do Norte Fluminense Darcy Ribeiro, Av. Alberto Lamego, 2000 – Parque Califórnia – CEP: 28013-602, Campos dos Goytacazes, Rio de Janeiro, Brazil

^b^ Departamento de Ciências Exatas, Universidade Estadual de Feira de Santana, Feira de Santana, Bahia, Brazil

^c^ Laboratório de Ciências Químicas, Centro de Ciência e Tecnologia, Universidade Estadual do Norte Fluminense Darcy Ribeiro, Av. Alberto Lamego, 2000 – Parque Califórnia – CEP: 28013-602, Campos dos Goytacazes, Rio de Janeiro, Brazil

*Corresponding author: luisaviana@pq.uenf.br

**Table S1.** Descriptive statistics of precipitation and monthly river discharge for the full record and contrasting hydroclimatic periods. Precipitation is expressed in mm and river discharge in m³ s⁻¹. Pre-2012 and Post-2012 denote the hydroclimatic periods used to characterize changes in discharge conditions.

| **Statistic** | **Precipitation** | **Flow – full record** | **Flow – Pre-2012** | **Flow – Post-2012** |
| --- | --- | --- | --- | --- |
| N | 631 | 634 | 547 | 87 |
| Mean | 158.87 | 5.41 | 5.52 | 4.71 |
| Median | 145.00 | 4.76 | 4.83 | 4.20 |
| Standard deviation | 90.00 | 2.90 | 2.92 | 2.66 |
| Minimum | 4.40 | 0.48 | 0.84 | 0.48 |
| Maximum | 654.50 | 22.16 | 22.16 | 14.35 |
| Coefficient of variation | 0.57 | 0.54 | 0.53 | 0.57 |

**Table S2.** Hyperparameters evaluated during the primary selection of multilayer perceptron models.

| **Component** | **Specification** |
| --- | --- |
| Network type | Feedforward multilayer perceptron |
| Software implementation | nnet package in R |
| Hidden layers | 1 (fixed) |
| Hidden-layer activation | Logistic sigmoid (fixed) |
| Output activation | Linear (fixed, linout = TRUE) |
| Training objective | Squared error |
| Optimization algorithm | BFGS |
| Hidden neurons evaluated | 1, 2, 3, 4, 5, 6, 8, 10 |
| Weight decay evaluated | 0, 0.0001, 0.001, 0.01 |
| Regularization | L2 weight decay controlled by the decay parameter |
| Maximum iterations | 1,000 |
| Early stopping | Not used |
| Random initializations per CV fold | 3 |
| Final-model random initializations | 20 |
| Random-seed control | Fixed base seed of 123 with deterministic offsets |
| Predictor and response scaling | z-score standardization using training data only |
| Cross-validation scheme | Rolling-origin cross-validation with expanding training window |
| Minimum initial training window | 20 years |
| Candidate origins | Final 120 months of each calibration period, advanced at 3-month intervals |
| Prediction horizon during CV | 1 month |
| Maximum CV folds | 40 for T1 and 39 for T2 |
| Primary selection criterion | Cross-validated RMSE |
| Complementary criteria | Cross-validated MAE and successful-fold proportion |
| External validation used for tuning | No |
| Frequency parameter | Not used in the MLP specification |
| Differencing parameter | Not used in the MLP specification |
| Selected T1 univariate model | U3: Q1, Q2, Q3; 3-8-1; decay = 0 |
| Selected T2 univariate model | U5: Q1, Q2, Q3, Q12; 4-4-1; decay = 0.01 |
| Selected T1 precipitation-informed model | M3: Q1, Q2, P1, P2; 4-4-1; decay = 0.01 |
| Selected T2 precipitation-informed model | M4: Q1, Q2, Q12, P1, P2; 5-10-1; decay = 0.001 |

**Table S3.** Accuracy statistics of ARMA candidate models under T1 and T2 temporal configurations. Only the 10 highest-ranked candidate models according to CV RMSE are shown for each temporal configuration. All eligible ARMA configurations were evaluated during model selection. CV MAE was used as a secondary ranking criterion.

| **Period** | **Class** | **p** | **d** | **q** | **CV RMSE** | **CV MAE** | **CV N** | **CV total** | **CV success rate (%)** |
| --- | --- | --- | --- | --- | --- | --- | --- | --- | --- |
| **T1** | **ARMA** | **1** | **0** | **2** | **1.9570** | **1.3685** | **40** | **40** | **100** |
| T1 | ARMA | 4 | 0 | 3 | 1.9718 | 1.3568 | 40 | 40 | 100 |
| T1 | ARMA | 3 | 0 | 4 | 1.9808 | 1.3402 | 40 | 40 | 100 |
| T1 | ARMA | 4 | 0 | 0 | 1.9813 | 1.3636 | 40 | 40 | 100 |
| T1 | ARMA | 1 | 0 | 3 | 1.9854 | 1.3843 | 40 | 40 | 100 |
| T1 | ARMA | 3 | 0 | 0 | 1.9855 | 1.3673 | 40 | 40 | 100 |
| T1 | ARMA | 2 | 0 | 2 | 1.9875 | 1.3858 | 40 | 40 | 100 |
| T1 | ARMA | 3 | 0 | 3 | 1.9888 | 1.3869 | 40 | 40 | 100 |
| T1 | ARMA | 2 | 0 | 4 | 1.9890 | 1.3881 | 40 | 40 | 100 |
| T1 | ARMA | 1 | 0 | 4 | 1.9891 | 1.3880 | 40 | 40 | 100 |
| **T2** | **ARMA** | **1** | **0** | **2** | **1.8681** | **1.2742** | **39** | **39** | **100** |
| T2 | ARMA | 3 | 0 | 1 | 1.8705 | 1.2715 | 39 | 39 | 100 |
| T2 | ARMA | 4 | 0 | 0 | 1.8707 | 1.2704 | 39 | 39 | 100 |
| T2 | ARMA | 4 | 0 | 3 | 1.8708 | 1.2599 | 39 | 39 | 100 |
| T2 | ARMA | 3 | 0 | 0 | 1.8718 | 1.2713 | 39 | 39 | 100 |
| T2 | ARMA | 4 | 0 | 1 | 1.8796 | 1.2860 | 39 | 39 | 100 |
| T2 | ARMA | 3 | 0 | 2 | 1.8800 | 1.2782 | 39 | 39 | 100 |
| T2 | ARMA | 1 | 0 | 3 | 1.8809 | 1.2847 | 39 | 39 | 100 |
| T2 | ARMA | 2 | 0 | 4 | 1.8826 | 1.2872 | 39 | 39 | 100 |
| T2 | ARMA | 1 | 0 | 4 | 1.8845 | 1.2856 | 39 | 39 | 100 |

**Table S4.** Accuracy statistics of ARIMA candidate models under T1 and T2 temporal configurations. Only the 10 highest-ranked candidate models according to CV RMSE are shown for each temporal configuration. All eligible ARIMA configurations were evaluated during model selection. CV MAE was used as a secondary ranking criterion.

| **Period** | **Class** | **p** | **d** | **q** | **CV RMSE** | **CV MAE** | **CV N** | **CV total** | **CV success rate (%)** |
| --- | --- | --- | --- | --- | --- | --- | --- | --- | --- |
| **T1** | **ARIMA** | **1** | **1** | **3** | **1.9565** | **1.3291** | **40** | **40** | **100** |
| T1 | ARIMA | 5 | 1 | 3 | 1.9579 | 1.3000 | 40 | 40 | 100 |
| T1 | ARIMA | 5 | 1 | 0 | 1.9607 | 1.3777 | 40 | 40 | 100 |
| T1 | ARIMA | 5 | 1 | 2 | 1.9608 | 1.3304 | 40 | 40 | 100 |
| T1 | ARIMA | 3 | 1 | 2 | 1.9616 | 1.3254 | 40 | 40 | 100 |
| T1 | ARIMA | 4 | 1 | 1 | 1.9628 | 1.3251 | 40 | 40 | 100 |
| T1 | ARIMA | 1 | 1 | 4 | 1.9628 | 1.3625 | 40 | 40 | 100 |
| T1 | ARIMA | 2 | 1 | 3 | 1.9636 | 1.3516 | 40 | 40 | 100 |
| T1 | ARIMA | 5 | 1 | 1 | 1.9646 | 1.3255 | 40 | 40 | 100 |
| T1 | ARIMA | 4 | 1 | 0 | 1.9659 | 1.3783 | 40 | 40 | 100 |
| **T2** | **ARIMA** | **2** | **1** | **4** | **1.8783** | **1.2864** | **39** | **39** | **100** |
| T2 | ARIMA | 1 | 1 | 3 | 1.8825 | 1.2904 | 39 | 39 | 100 |
| T2 | ARIMA | 1 | 1 | 4 | 1.8841 | 1.2909 | 39 | 39 | 100 |
| T2 | ARIMA | 5 | 1 | 2 | 1.8888 | 1.2914 | 39 | 39 | 100 |
| T2 | ARIMA | 2 | 1 | 3 | 1.8901 | 1.2897 | 39 | 39 | 100 |
| T2 | ARIMA | 3 | 1 | 4 | 1.8950 | 1.2985 | 39 | 39 | 100 |
| T2 | ARIMA | 4 | 1 | 3 | 1.8952 | 1.3043 | 39 | 39 | 100 |
| T2 | ARIMA | 4 | 1 | 1 | 1.8999 | 1.3023 | 39 | 39 | 100 |
| T2 | ARIMA | 3 | 1 | 2 | 1.9021 | 1.3065 | 39 | 39 | 100 |
| T2 | ARIMA | 5 | 1 | 1 | 1.9039 | 1.3039 | 39 | 39 | 100 |
| T2 | ARIMA | 4 | 1 | 2 | 1.9046 | 1.3044 | 39 | 39 | 100 |

**Table S5.** Accuracy statistics of SARIMA candidate models under T1 and T2 temporal configurations. Only the 10 highest-ranked candidate models according to CV RMSE are shown for each temporal configuration. All eligible SARIMA configurations were evaluated during model selection. CV MAE was used as a secondary ranking criterion.

| Period | Class | p | d | q | P | D | Q | s | CV RMSE | CV MAE | CV N | CV total | CV success |
| --- | --- | --- | --- | --- | --- | --- | --- | --- | --- | --- | --- | --- | --- |
| **T1** | **SARIMA** | **2** | **1** | **2** | **2** | **0** | **0** | **12** | **1.9112** | **1.3152** | **38** | **40** | **0.950** |
| T1 | SARIMA | 1 | 1 | 3 | 0 | 0 | 2 | 12 | 1.9528 | 1.3183 | 40 | 40 | 1 |
| T1 | SARIMA | 1 | 1 | 3 | 0 | 0 | 0 | 12 | 1.9565 | 1.3291 | 40 | 40 | 1 |
| T1 | SARIMA | 1 | 1 | 3 | 0 | 0 | 1 | 12 | 1.9587 | 1.3225 | 40 | 40 | 1 |
| T1 | SARIMA | 3 | 1 | 2 | 0 | 0 | 0 | 12 | 1.9616 | 1.3254 | 40 | 40 | 1 |
| T1 | SARIMA | 2 | 1 | 3 | 0 | 0 | 0 | 12 | 1.9636 | 1.3516 | 40 | 40 | 1 |
| T1 | SARIMA | 1 | 1 | 3 | 1 | 0 | 0 | 12 | 1.9668 | 1.3153 | 39 | 40 | 0.975 |
| T1 | SARIMA | 3 | 1 | 1 | 0 | 0 | 0 | 12 | 1.9674 | 1.3435 | 40 | 40 | 1 |
| T1 | SARIMA | 3 | 1 | 1 | 1 | 0 | 2 | 12 | 1.9709 | 1.3326 | 40 | 40 | 1 |
| T1 | SARIMA | 3 | 1 | 2 | 0 | 0 | 2 | 12 | 1.9728 | 1.3273 | 40 | 40 | 1 |
| **T2** | **SARIMA** | **3** | **1** | **2** | **1** | **0** | **1** | **12** | **1.3094** | **1.0918** | **38** | **39** | **0.974** |
| T2 | SARIMA | 1 | 1 | 3 | 1 | 0 | 1 | 12 | 1.8705 | 1.2707 | 39 | 39 | 1 |
| T2 | SARIMA | 1 | 1 | 3 | 0 | 0 | 2 | 12 | 1.8739 | 1.2699 | 39 | 39 | 1 |
| T2 | SARIMA | 1 | 1 | 3 | 1 | 0 | 2 | 12 | 1.8784 | 1.2234 | 36 | 39 | 0.923 |
| T2 | SARIMA | 1 | 1 | 3 | 1 | 0 | 0 | 12 | 1.8812 | 1.277 | 39 | 39 | 1 |
| T2 | SARIMA | 1 | 1 | 3 | 0 | 0 | 1 | 12 | 1.8818 | 1.2773 | 39 | 39 | 1 |
| T2 | SARIMA | 1 | 1 | 3 | 0 | 0 | 0 | 12 | 1.8825 | 1.2904 | 39 | 39 | 1 |
| T2 | SARIMA | 3 | 1 | 2 | 1 | 0 | 2 | 12 | 1.8866 | 1.2829 | 39 | 39 | 1 |
| T2 | SARIMA | 3 | 1 | 2 | 0 | 0 | 2 | 12 | 1.8894 | 1.2805 | 39 | 39 | 1 |
| T2 | SARIMA | 2 | 1 | 3 | 0 | 0 | 0 | 12 | 1.8901 | 1.2897 | 39 | 39 | 1 |

**Table S6.** Rolling-origin cross-validation performance of candidate univariate MLP models under the T1 and T2 temporal configurations. Only the 10 highest-ranked models according to CV RMSE are shown for each configuration. All eligible univariate MLP configurations were evaluated during model selection, with CV MAE used as a secondary ranking criterion. Bold indicates the model selected for each temporal configuration.

| **Period** | **Type** | **Model** | **Predictors** | **Hidden** | **Decay** | **Architecture** | **CV RMSE** | **CV MAE** | **CV N** | **CV total** | **CV success rate (%)** |
| --- | --- | --- | --- | --- | --- | --- | --- | --- | --- | --- | --- |
| **T1** | **Univariate** | **U3** | **Q1+Q2+Q3** | **8** | **0.0000** | **3-8-1** | **1.7132** | **1.1609** | **40** | **40** | **100** |
| T1 | Univariate | U3 | Q1+Q2+Q3 | 10 | 0.0000 | 3-10-1 | 1.7239 | 1.2091 | 40 | 40 | 100 |
| T1 | Univariate | U3 | Q1+Q2+Q3 | 6 | 0.01 | 3-6-1 | 1.7570 | 1.1253 | 40 | 40 | 100 |
| T1 | Univariate | U5 | Q1+Q2+Q3+Q12 | 4 | 0.01 | 4-4-1 | 1.7605 | 1.2629 | 40 | 40 | 100 |
| T1 | Univariate | U3 | Q1+Q2+Q3 | 8 | 0.01 | 3-8-1 | 1.7868 | 1.1303 | 40 | 40 | 100 |
| T1 | Univariate | U3 | Q1+Q2+Q3 | 6 | 0.0001 | 3-6-1 | 1.8069 | 1.2451 | 40 | 40 | 100 |
| T1 | Univariate | U3 | Q1+Q2+Q3 | 6 | 0.0000 | 3-6-1 | 1.8139 | 1.2222 | 40 | 40 | 100 |
| T1 | Univariate | U3 | Q1+Q2+Q3 | 8 | 0.001 | 3-8-1 | 1.8209 | 1.2089 | 40 | 40 | 100 |
| T1 | Univariate | U2 | Q1+Q2 | 3 | 0.0000 | 2-3-1 | 1.8413 | 1.3014 | 40 | 40 | 100 |
| T1 | Univariate | U3 | Q1+Q2+Q3 | 8 | 0.0001 | 3-8-1 | 1.8421 | 1.2958 | 40 | 40 | 100 |
| **T2** | **Univariate** | **U5** | **Q1+Q2+Q3+Q12** | **4** | **0.01** | **4-4-1** | **1.7256** | **1.2313** | **37** | **37** | **100** |
| T2 | Univariate | U2 | Q1+Q2 | 3 | 0.0000 | 2-3-1 | 1.7782 | 1.2951 | 39 | 39 | 100 |
| T2 | Univariate | U2 | Q1+Q2 | 8 | 0.0000 | 2-8-1 | 1.8419 | 1.2501 | 39 | 39 | 100 |
| T2 | Univariate | U3 | Q1+Q2+Q3 | 8 | 0.0000 | 3-8-1 | 1.8438 | 1.3054 | 38 | 38 | 100 |
| T2 | Univariate | U2 | Q1+Q2 | 3 | 0.0001 | 2-3-1 | 1.8459 | 1.3023 | 39 | 39 | 100 |
| T2 | Univariate | U3 | Q1+Q2+Q3 | 3 | 0.01 | 3-3-1 | 1.8491 | 1.2666 | 38 | 38 | 100 |
| T2 | Univariate | U2 | Q1+Q2 | 3 | 0.001 | 2-3-1 | 1.8499 | 1.3115 | 39 | 39 | 100 |
| T2 | Univariate | U3 | Q1+Q2+Q3 | 2 | 0.0000 | 3-2-1 | 1.8529 | 1.2340 | 38 | 38 | 100 |
| T2 | Univariate | U3 | Q1+Q2+Q3 | 2 | 0.001 | 3-2-1 | 1.8539 | 1.2327 | 38 | 38 | 100 |
| T2 | Univariate | U3 | Q1+Q2+Q3 | 2 | 0.0001 | 3-2-1 | 1.8550 | 1.2336 | 38 | 38 | 100 |

**Table S7.** Rolling-origin cross-validation performance of candidate multivariate MLP models under the T1 and T2 temporal configurations. Only the 10 highest-ranked models according to CV RMSE are shown for each configuration. All eligible multivariate MLP configurations were evaluated during model selection, with CV MAE used as a secondary ranking criterion. Bold indicates the model selected for each temporal configuration.

| Period | Type | Model | Predictors | Hidden | Decay | Architecture | CV RMSE | CV MAE | CV N | CV total | CV success rate (%) |
| --- | --- | --- | --- | --- | --- | --- | --- | --- | --- | --- | --- |
| **T1** | **Multivariate** | **M3** | **Q1+Q2+P1+P2** | **4** | **0.01** | **4-4-1** | **1.7942** | **1.2102** | **40** | **40** | **100** |
| T1 | Multivariate | M3 | Q1+Q2+P1+P2 | 4 | 0.0001 | 4-4-1 | 1.8040 | 1.2543 | 40 | 40 | 100 |
| T1 | Multivariate | M6 | Q1+Q2+Q3+P1+P2 | 10 | 0.001 | 5-10-1 | 1.8095 | 1.3439 | 40 | 40 | 100 |
| T1 | Multivariate | M4 | Q1+Q2+Q12+P1+P2 | 10 | 0.001 | 5-10-1 | 1.8109 | 1.2871 | 40 | 40 | 100 |
| T1 | Multivariate | M3 | Q1+Q2+P1+P2 | 4 | 0.001 | 4-4-1 | 1.8124 | 1.2622 | 40 | 40 | 100 |
| T1 | Multivariate | M6 | Q1+Q2+Q3+P1+P2 | 6 | 0.001 | 5-6-1 | 1.8146 | 1.1867 | 40 | 40 | 100 |
| T1 | Multivariate | M3 | Q1+Q2+P1+P2 | 4 | 0.0000 | 4-4-1 | 1.8214 | 1.2829 | 40 | 40 | 100 |
| T1 | Multivariate | M3 | Q1+Q2+P1+P2 | 6 | 0.01 | 4-6-1 | 1.8222 | 1.2476 | 40 | 40 | 100 |
| T1 | Multivariate | M3 | Q1+Q2+P1+P2 | 6 | 0.001 | 4-6-1 | 1.8329 | 1.2499 | 40 | 40 | 100 |
| T1 | Multivariate | M6 | Q1+Q2+Q3+P1+P2 | 3 | 0.0000 | 5-3-1 | 1.8389 | 1.3118 | 40 | 40 | 100 |
| **T2** | **Multivariate** | **M4** | **Q1+Q2+Q12+P1+P2** | **10** | **0.001** | **5-10-1** | **1.7241** | **1.2831** | **37** | **37** | **100** |
| T2 | Multivariate | M7 | Q1+Q2+Q3+Q12+P1+P2 | 10 | 0.01 | 6-10-1 | 1.8332 | 1.2970 | 36 | 36 | 100 |
| T2 | Multivariate | M1 | Q1+P1 | 5 | 0.0000 | 2-5-1 | 1.8405 | 1.2613 | 39 | 39 | 100 |
| T2 | Multivariate | M4 | Q1+Q2+Q12+P1+P2 | 6 | 0.0001 | 5-6-1 | 1.8462 | 1.2777 | 37 | 37 | 100 |
| T2 | Multivariate | M2 | Q1+Q2+P1 | 8 | 0.01 | 3-8-1 | 1.8622 | 1.3026 | 39 | 39 | 100 |
| T2 | Multivariate | M2 | Q1+Q2+P1 | 6 | 0.01 | 3-6-1 | 1.8624 | 1.3032 | 39 | 39 | 100 |
| T2 | Multivariate | M1 | Q1+P1 | 10 | 0.0000 | 2-10-1 | 1.8624 | 1.2886 | 39 | 39 | 100 |
| T2 | Multivariate | M4 | Q1+Q2+Q12+P1+P2 | 6 | 0.001 | 5-6-1 | 1.8629 | 1.2862 | 37 | 37 | 100 |
| T2 | Multivariate | M2 | Q1+Q2+P1 | 3 | 0.01 | 3-3-1 | 1.8655 | 1.3081 | 39 | 39 | 100 |
| T2 | Multivariate | M1 | Q1+P1 | 4 | 0.0000 | 2-4-1 | 1.8728 | 1.3033 | 39 | 39 | 100 |

**Table S8.** Rolling-origin cross-validation performance of candidate multiple linear regression models under the T1 and T2 temporal configurations. Only the 10 highest-ranked models according to CV RMSE are shown for each configuration. All eligible predictor combinations were evaluated during model selection, with CV MAE used as a secondary ranking criterion. Bold indicates the model selected for each temporal configuration.

| Period | Model | Predictors | CV RMSE | CV MAE | CV N | CV total | CV success rate (%) |
| --- | --- | --- | --- | --- | --- | --- | --- |
| **T1** | **M6** | **Q1+Q2+Q3+P1+P2** | **1.9583** | **1.3573** | **40** | **40** | **100** |
| T1 | M7 | Q1+Q2+Q3+Q12+P1+P2 | 1.9910 | 1.3841 | 40 | 40 | 100 |
| T1 | M3 | Q1+Q2+P1+P2 | 2.0104 | 1.3892 | 40 | 40 | 100 |
| T1 | M2 | Q1+Q2+P1 | 2.0206 | 1.3883 | 40 | 40 | 100 |
| T1 | M1 | Q1+P1 | 2.0369 | 1.4068 | 40 | 40 | 100 |
| T1 | M4 | Q1+Q2+Q12+P1+P2 | 2.0530 | 1.4366 | 40 | 40 | 100 |
| T1 | M8 | Q1+Q2+Q12+P1+P2+P12 | 2.0566 | 1.4431 | 40 | 40 | 100 |
| T1 | M5 | Q1+Q2+Q12+P1+P12 | 2.0635 | 1.4258 | 40 | 40 | 100 |
| **T2** | **M1** | **Q1+P1** | **1.9179** | **1.2667** | **39** | **39** | **100** |
| T2 | M2 | Q1+Q2+P1 | 1.9227 | 1.2781 | 39 | 39 | 100 |
| T2 | M6 | Q1+Q2+Q3+P1+P2 | 1.9317 | 1.3086 | 37 | 37 | 100 |
| T2 | M5 | Q1+Q2+Q12+P1+P12 | 1.9382 | 1.2473 | 38 | 38 | 100 |
| T2 | M3 | Q1+Q2+P1+P2 | 1.9506 | 1.3198 | 38 | 38 | 100 |
| T2 | M7 | Q1+Q2+Q3+Q12+P1+P2 | 1.9643 | 1.3037 | 36 | 36 | 100 |
| T2 | M8 | Q1+Q2+Q12+P1+P2+P12 | 1.9654 | 1.2950 | 37 | 37 | 100 |
| T2 | M4 | Q1+Q2+Q12+P1+P2 | 1.9854 | 1.3162 | 37 | 37 | 100 |

**Table S9.** Tests of temporal change in the Serinhaém River discharge series. Panel A presents structural-change and trend tests applied to annual discharge from 1966 to 2018. Estimated candidate change points correspond to the positions maximizing the respective test statistics but do not represent statistically confirmed breakpoints. Panel B presents direct comparisons of monthly discharge between the pre-2012 period (January 1966–December 2011) and the post-2012 period (January 2012–April 2019). Panel B p-values were adjusted across the four comparisons using the Benjamini–Hochberg procedure. Sen’s slope is expressed in m³ s⁻¹ yr⁻¹. The asterisk denotes statistical significance at BH-adjusted p < 0.05.

| **Panel A. Annual structural-change and trend tests** | | | | |
| --- | --- | --- | --- | --- |
| **Test** | **Statistic** | **Estimate** | **p-value** | **Estimated candidate change point** |
| Pettitt | U* = 276 | — | 0.0824 | K = 20 (1985) |
| Buishand U | U = 0.393 | — | 0.0726 | K = 20 (1985) |
| SNHT | T = 7.262 | — | 0.1035 | K = 16 (1981) |
| Mann–Kendall | Z = −1.444 | — | 0.1487 | — |
| Sen’s slope | Z = −1.444 | β = −0.0232 m³ s⁻¹ yr⁻¹ | 0.1487 | — |
| **Panel B. Direct comparisons between pre-2012 and post-2012 monthly discharge** | | | | |
| **Test** | **Statistic** | **Raw p-value** | **BH-adjusted p-value** | **Result** |
| Welch mean comparison | t = 2.618 | 0.0100 | **0.0200*** | Lower mean after 2012 |
| Wilcoxon location comparison | W = 27,983 | 0.0083 | **0.0200*** | Lower distributional location after 2012 |
| Brown–Forsythe variance comparison | F = 0.269 | 0.6043 | 0.8057 | No significant variance difference |
| Month × period interaction | F = 0.450 | 0.9327 | 0.9327 | No significant month-by-period interaction |

* Estimated change points indicate the locations of the maximum test statistics. Because the corresponding tests were not significant at α = 0.05, these locations should not be interpreted as statistically supported breakpoints.

**Figure S1.** (A) Monthly freshwater discharge of the Serinhaém River, Bahia, Brazil, from January 1966 to April 2019; (B) autocorrelation function (ACF); and (C) partial autocorrelation function (PACF) of the monthly discharge series. Dashed lines indicate the approximate 95% confidence limits for zero autocorrelation.

**Figure S2.** Monthly freshwater discharge of the Serinhaém River, Bahia, Brazil, from January 1966 to April 2019, showing the temporal partitions used in the T1 and T2 configurations. The vertical gray dashed line indicates the beginning of the T1 validation period in January 2006, whereas the vertical red solid line indicates the beginning of the T2 validation period in January 2012. The horizontal red and blue dotted lines represent, respectively, the 10th and 90th percentiles of the observed discharge series.

**Table S10.** Spearman rank correlations between monthly discharge and contemporaneous and lagged monthly precipitation. *N* denotes the number of paired observations available at each lag. *P*-values were adjusted for multiple comparisons using the Benjamini–Hochberg procedure. Lag 0 represents contemporaneous precipitation, whereas positive lags represent precipitation occurring before the corresponding discharge observation.

| Precipitation_lag | N | Spearman_rho | P_value | P_adjusted_BH |
| --- | --- | --- | --- | --- |
| 0 | 628 | 0.434 | 2.80 × 10⁻³⁰ | 1.40 × 10⁻²⁹ |
| 1 | 626 | 0.401 | 1.45 × 10⁻²⁵ | 3.62 × 10⁻²⁵ |
| 2 | 624 | 0.296 | 4.26 × 10⁻¹⁴ | 7.09 × 10⁻¹⁴ |
| 3 | 622 | 0.280 | 1.16 × 10⁻¹² | 1.45 × 10⁻¹² |
| 12 | 615 | 0.146 | 2.71 × 10⁻⁴ | 2.71 × 10⁻⁴ |

**Figure S3.** Relationship between monthly precipitation and river discharge and their temporal cross-correlation. **(A)** Monthly precipitation versus observed monthly discharge. Points represent individual monthly observations, and the blue curve shows the LOESS-smoothed relationship with its 95% confidence band; the contemporaneous Spearman correlation is also indicated. **(B)** Cross-correlation function (CCF) between monthly precipitation and discharge for lags from −24 to +24 months. Negative lags indicate precipitation leading discharge, whereas positive lags indicate discharge leading precipitation. Dashed horizontal lines represent the approximate 95% significance limits.

**Table S11.** Independent-validation performance of the selected univariate and multivariate MLP configurations calculated using identical prediction dates under T1 and T2. Positive differences denote higher error for the multivariate configuration relative to the univariate configuration. RMSE and MAE are expressed in m³ s⁻¹. Differences (Δ) were calculated as multivariate minus univariate MLP; positive values indicate higher error for the multivariate model.

| **Period** | **N common** | **RMSE univariate** | **RMSE multivariate** | **MAE univariate** | **MAE multivariate** | **ΔRMSE** | **ΔMAE** |
| --- | --- | --- | --- | --- | --- | --- | --- |
| T1 | 143 | 1.7641 | 1.8007 | 1.3063 | 1.3059 | 0.0366 | −0.0004 |
| T2 | 77 | 1.8378 | 1.8914 | 1.2997 | 1.2891 | 0.0536 | -0.0106 |

**Table S12.** Flow-dependent performance of the selected multilayer perceptron (MLP) models during pseudo-out-of-sample calibration and independent validation under the T1 and T2 temporal configurations. Calibration results were obtained from one-step pseudo-out-of-sample predictions generated through the rolling-origin procedure, whereas validation results correspond to the independent held-out periods. Observed discharge was divided into low, intermediate, and high-flow classes using terciles defined separately within each temporal configuration and evaluation stage. RMSE, MAE, mean prediction bias, and the fraction of observations for which discharge was underpredicted are reported for each flow class. Positive bias indicates average overprediction and negative bias indicates average underprediction. RMSE, MAE, and bias are expressed in m³ s⁻¹.

| Period | Family | Model | Architecture | Flow class | N | RMSE | MAE | Bias | Underprediction fraction | Stage |
| --- | --- | --- | --- | --- | --- | --- | --- | --- | --- | --- |
| T1 | MLP univariate | U3 | 3-8-1 | Low | 117 | 1,7830 | 1,4399 | 1,3461 | 0,1026 | Calibration pseudo-out-of-sample |
| T1 | MLP univariate | U3 | 3-8-1 | Intermediate | 115 | 1,5814 | 1,2700 | 0,5342 | 0,3652 | Calibration pseudo-out-of-sample |
| T1 | MLP univariate | U3 | 3-8-1 | High | 115 | 3,8266 | 2,4712 | -1,7053 | 0,7304 | Calibration pseudo-out-of-sample |
| T1 | MLP univariate | U3 | 3-8-1 | Low | 51 | 1,6537 | 1,3298 | 1,2204 | 0,1176 | Independent validation |
| T1 | MLP univariate | U3 | 3-8-1 | Intermediate | 51 | 1,1570 | 0,9765 | 0,5966 | 0,2941 | Independent validation |
| T1 | MLP univariate | U3 | 3-8-1 | High | 50 | 2,2240 | 1,5367 | -0,8599 | 0,6400 | Independent validation |
| T1 | MLP multivariate | M3 | 4-4-1 | Low | 117 | 1,6177 | 1,3811 | 1,3595 | 0,0513 | Calibration pseudo-out-of-sample |
| T1 | MLP multivariate | M3 | 4-4-1 | Intermediate | 117 | 1,4954 | 1,1365 | 0,4622 | 0,4274 | Calibration pseudo-out-of-sample |
| T1 | MLP multivariate | M3 | 4-4-1 | High | 116 | 3,2994 | 2,2746 | -1,6189 | 0,7155 | Calibration pseudo-out-of-sample |
| T1 | MLP multivariate | M3 | 4-4-1 | Low | 48 | 1,4089 | 1,1768 | 1,1158 | 0,0625 | Independent validation |
| T1 | MLP multivariate | M3 | 4-4-1 | Intermediate | 48 | 1,2032 | 0,9815 | 0,6471 | 0,2500 | Independent validation |
| T1 | MLP multivariate | M3 | 4-4-1 | High | 49 | 2,4735 | 1,7240 | -1,2345 | 0,7551 | Independent validation |
| T2 | MLP univariate | U5 | 4-4-1 | Low | 138 | 1,5898 | 1,3330 | 1,3121 | 0,0362 | Calibration pseudo-out-of-sample |
| T2 | MLP univariate | U5 | 4-4-1 | Intermediate | 135 | 1,3724 | 1,1124 | 0,5749 | 0,2963 | Calibration pseudo-out-of-sample |
| T2 | MLP univariate | U5 | 4-4-1 | High | 137 | 3,2225 | 2,2369 | -1,2645 | 0,6496 | Calibration pseudo-out-of-sample |
| T2 | MLP univariate | U5 | 4-4-1 | Low | 28 | 1,3209 | 1,0528 | 0,9591 | 0,1071 | Independent validation |
| T2 | MLP univariate | U5 | 4-4-1 | Intermediate | 28 | 1,1755 | 1,0046 | 0,3545 | 0,4286 | Independent validation |
| T2 | MLP univariate | U5 | 4-4-1 | High | 28 | 2,5422 | 1,7467 | -0,9199 | 0,6429 | Independent validation |
| T2 | MLP multivariate | M4 | 5-10-1 | Low | 137 | 1,8081 | 1,4546 | 1,3487 | 0,1314 | Calibration pseudo-out-of-sample |
| T2 | MLP multivariate | M4 | 5-10-1 | Intermediate | 137 | 1,6042 | 1,2139 | 0,4960 | 0,3869 | Calibration pseudo-out-of-sample |
| T2 | MLP multivariate | M4 | 5-10-1 | High | 138 | 3,3242 | 2,3875 | -1,4490 | 0,6957 | Calibration pseudo-out-of-sample |
| T2 | MLP multivariate | M4 | 5-10-1 | Low | 25 | 1,3882 | 1,0869 | 1,0227 | 0,0400 | Independent validation |
| T2 | MLP multivariate | M4 | 5-10-1 | Intermediate | 27 | 1,2216 | 0,9913 | 0,4864 | 0,2963 | Independent validation |
| T2 | MLP multivariate | M4 | 5-10-1 | High | 26 | 2,6910 | 1,7866 | -1,2163 | 0,7308 | Independent validation |

**Figure S4.** Absolute one-step prediction error as a function of observed monthly discharge for the selected predictive models under the T1 and T2 temporal configurations. Each point represents one validation month. Red curves show the fitted smooth relationship between absolute error and observed discharge, and grey bands represent the associated 95% confidence intervals. Absolute errors were generally lowest at intermediate discharge values and increased toward high-flow events across model families.

**Table S13.** Predictor-matched comparison of linear regression and multilayer perceptron (MLP) models using identical input variables and validation dates under the T1 and T2 temporal configurations. Differences (Δ) were calculated as MLP minus linear regression; therefore, positive values indicate higher errors for the MLP. The 95% confidence intervals for differences in MAE were estimated using a 12-month moving-block bootstrap with 2,000 replicates. Diebold–Mariano p-values were adjusted across the four comparisons using the Benjamini–Hochberg procedure. RMSE, MAE, ΔRMSE, and ΔMAE are expressed in m³ s⁻¹. The asterisk denotes statistical significance at adjusted p < 0.05.

| **Period** | **Information domain** | **Predictors** | **Selected MLP architecture** | **N** | **RMSE linear** | **RMSE MLP** | **MAE linear** | **MAE MLP** | **ΔRMSE** | **ΔMAE [95% CI]** | **DM** | **Adjusted p** |
| --- | --- | --- | --- | --- | --- | --- | --- | --- | --- | --- | --- | --- |
| T1 | Discharge + precipitation | Q1+Q2+Q3+P1+P2 | 5–10–1; decay = 0.001 | 143 | 1.6935 | 1.9478 | 1.2349 | 1.4557 | 0.2543 | 0.2208 [0.0976, 0.3438] | −3.4736 | **0.0027*** |
| T1 | Discharge only | Q1+Q2+Q3 | 3–8–1; decay = 0 | 152 | 1.6526 | 1.7302 | 1.2517 | 1.2793 | 0.0776 | 0.0276 [−0.0756, 0.1351] | −0.5043 | 0.8808 |
| T2 | Discharge + precipitation | Q1+Q2+Q3+P1+P2 | 5–3–1; decay = 0.0001 | 77 | 1.8470 | 1.8511 | 1.2557 | 1.2759 | 0.0041 | 0.0201 [−0.0725, 0.1125] | −0.4072 | 0.8808 |
| T2 | Discharge only | Q1+Q2+Q3 | 3–8–1; decay = 0 | 84 | 1.8354 | 1.8991 | 1.3333 | 1.3221 | 0.0637 | −0.0112 [−0.1562, 0.1429] | 0.1504 | 0.8808 |

**Table S14.** Independent one-step and recursive validation performance of the selected benchmark, linear regression, stochastic, and multilayer perceptron (MLP) models under the T1 and T2 temporal configurations. *One-step* denotes predictions generated using observed antecedent discharge values, whereas *Recursive* denotes predictions generated using previous model predictions as inputs when required. Metrics were calculated using all valid predictions available for each model and evaluation strategy. Because the number and dates of valid predictions differed among models and temporal configurations, comparisons involving different sample sizes should be interpreted descriptively. RMSE, MAE, and Bias are expressed in m³ s⁻¹; PBIAS is expressed as a percentage; and NSE, KGE_2012_, RSR, MASE, R², and relative variance error are dimensionless.

| Period | Family | Model | Evaluation | N | RMSE | MAE | NSE | KGE_2012_ | PBIAS | RSR | MASE | R^2^ | Bias | Relative variance error |
| --- | --- | --- | --- | --- | --- | --- | --- | --- | --- | --- | --- | --- | --- | --- |
| T1 | ARMA | ARMA(1,0,2)(0,0,0)[12] | One-step | 158 | 1.6357 | 1.2358 | 0.5046 | 0.5123 | 5.8232 | 0.7016 | 0.4355 | 0.5269 | 0.2767 | -0.5958 |
| T1 | ARIMA | ARIMA(1,1,3)(0,0,0)[12] | One-step | 158 | 1.6363 | 1.2001 | 0.5043 | 0.5301 | 3.0272 | 0.7019 | 0.4229 | 0.5131 | 0.1438 | -0.5832 |
| T1 | SARIMA | SARIMA(2,1,2)(2,0,0)[12] | One-step | 158 | 1.6563 | 1.2033 | 0.4921 | 0.5246 | 2.4100 | 0.7104 | 0.4240 | 0.4986 | 0.1145 | -0.5876 |
| T1 | Linear regression | Linear [M6] | One-step | 143 | 1.6935 | 1.2349 | 0.4836 | 0.5045 | 1.4090 | 0.7161 | 0.4352 | 0.4922 | 0.0675 | -0.6239 |
| T1 | MLP univariate | MLP univariate [U3; 3-8-1] | One-step | 152 | 1.7302 | 1.2793 | 0.4566 | 0.5584 | 6.8694 | 0.7347 | 0.4508 | 0.4783 | 0.3268 | -0.4538 |
| T1 | MLP multivariate | MLP multivariate [M3; 4-4-1] | One-step | 145 | 1.7900 | 1.2970 | 0.4213 | 0.5067 | 3.4820 | 0.7581 | 0.4571 | 0.4267 | 0.1664 | -0.5462 |
| T1 | Benchmark | Seasonal naive | One-step | 157 | 3.1446 | 2.2575 | -0.8391 | 0.0824 | 1.7781 | 1.3518 | 0.7955 | 0.0068 | 0.0841 | 0.0036 |
| T2 | MLP univariate | MLP univariate [U5; 4-4-1] | One-step | 84 | 1.7878 | 1.2680 | 0.5510 | 0.5977 | 2.7636 | 0.6661 | 0.4676 | 0.5546 | 0.1313 | -0.4968 |
| T2 | Linear regression | Linear [M1] | One-step | 82 | 1.8109 | 1.2688 | 0.5385 | 0.4841 | 2.5312 | 0.6752 | 0.4679 | 0.5840 | 0.1190 | -0.6914 |
| T2 | ARIMA | ARIMA(2,1,4)(0,0,0)[12] | One-step | 87 | 1.8242 | 1.3288 | 0.5257 | 0.5301 | 5.0323 | 0.6847 | 0.4901 | 0.5420 | 0.2369 | -0.5836 |
| T2 | SARIMA | SARIMA(3,1,2)(1,0,1)[12] | One-step | 87 | 1.8244 | 1.3144 | 0.5256 | 0.5298 | 4.8018 | 0.6848 | 0.4847 | 0.5414 | 0.2260 | -0.5859 |
| T2 | ARMA | ARMA(1,0,2)(0,0,0)[12] | One-step | 87 | 1.8274 | 1.3315 | 0.5241 | 0.5228 | 5.3804 | 0.6859 | 0.4911 | 0.5430 | 0.2533 | -0.5927 |
| T2 | MLP multivariate | MLP multivariate [M4; 5-10-1] | One-step | 78 | 1.8836 | 1.2871 | 0.5092 | 0.5519 | 1.9057 | 0.6961 | 0.4747 | 0.5128 | 0.0907 | -0.5561 |
| T2 | Benchmark | Seasonal naive | One-step | 87 | 3.5242 | 2.5597 | -0.7700 | 0.0932 | 0.9206 | 1.3228 | 0.9440 | 0.0088 | 0.0433 | -0.0470 |
| T1 | Linear regression | Linear [M6] | Recursive | 40 | 1.9945 | 1.5995 | 0.2021 | 0.1147 | 2.5840 | 0.8820 | 0.5637 | 0.2767 | 0.1310 | -0.9328 |
| T1 | ARMA | ARMA(1,0,2)(0,0,0)[12] | Recursive | 158 | 2.4820 | 2.0366 | -0.1406 | -0.4513 | 18.2822 | 1.0646 | 0.7177 | 0.0019 | 0.8686 | -0.9999 |
| T1 | SARIMA | SARIMA(2,1,2)(2,0,0)[12] | Recursive | 158 | 2.5309 | 2.0847 | -0.1859 | -0.4177 | 21.0911 | 1.0855 | 0.7346 | 0.0000 | 1.0021 | -0.9998 |
| T1 | Benchmark | Seasonal naive | Recursive | 158 | 2.5327 | 1.8761 | -0.1876 | 0.1741 | 10.3345 | 1.0863 | 0.6611 | 0.0671 | 0.4910 | -0.4853 |
| T1 | ARIMA | ARIMA(1,1,3)(0,0,0)[12] | Recursive | 158 | 2.5352 | 2.0899 | -0.1899 | -0.4244 | 21.3013 | 1.0874 | 0.7365 | 0.0000 | 1.0121 | -0.9998 |
| T1 | MLP multivariate | MLP multivariate [M3; 4-4-1] | Recursive | 40 | 2.5647 | 2.1918 | -0.3193 | -0.4300 | 16.6768 | 1.1341 | 0.7724 | 0.0374 | 0.8452 | -0.9279 |
| T1 | MLP univariate | MLP univariate [U3; 3-8-1] | Recursive | 158 | 3.8102 | 3.3770 | -1.6878 | -0.5200 | 63.3226 | 1.6343 | 1.1900 | 0.0002 | 3.0086 | -0.9905 |
| T2 | Linear regression | Linear [M1] | Recursive | 13 | 1.6055 | 1.3904 | -2.6987 | -0.2653 | 40.8347 | 1.8478 | 0.5128 | 0.0300 | 1.3790 | -0.9646 |
| T2 | MLP multivariate | MLP multivariate [M4; 5-10-1] | Recursive | 13 | 2.0650 | 1.8820 | -5.1191 | -0.2892 | 55.7310 | 2.3766 | 0.6941 | 0.0165 | 1.8820 | -0.8709 |
| T2 | SARIMA | SARIMA(3,1,2)(1,0,1)[12] | Recursive | 87 | 2.7272 | 2.1942 | -0.0599 | -0.3073 | 14.8290 | 1.0236 | 0.8092 | 0.0152 | 0.6980 | -0.9977 |
| T2 | Benchmark | Seasonal naive | Recursive | 87 | 2.7387 | 2.0534 | -0.0689 | -0.1958 | -3.1574 | 1.0279 | 0.7573 | 0.0010 | -0.1486 | -0.9160 |
| T2 | ARIMA | ARIMA(2,1,4)(0,0,0)[12] | Recursive | 87 | 2.7391 | 2.2058 | -0.0692 | -0.2930 | 15.9726 | 1.0281 | 0.8135 | 0.0228 | 0.7518 | -0.9981 |
| T2 | ARMA | ARMA(1,0,2)(0,0,0)[12] | Recursive | 87 | 2.7402 | 2.2075 | -0.0701 | -0.2935 | 16.0467 | 1.0285 | 0.8141 | 0.0229 | 0.7553 | -0.9981 |
| T2 | MLP univariate | MLP univariate [U5; 4-4-1] | Recursive | 87 | 3.4006 | 2.8813 | -0.6480 | -0.3253 | 46.1728 | 1.2763 | 1.0626 | 0.0282 | 2.1734 | -0.9871 |

**Figure S5.** Matched-period comparison of one-step predictive performance under the T1 and T2 temporal configurations using model-specific common prediction dates from January 2012 to April 2019. Panels show root mean square error (RMSE), mean absolute error (MAE), the ratio of RMSE to the standard deviation of observed discharge (RSR), mean absolute scaled error (MASE), and relative variance error. Within each model family, T1 and T2 metrics were calculated using exactly the same observed months. Relative variance error was calculated as [Var(prediction) − Var(observation)]/Var(observation); values close to zero indicate similar predicted and observed variability, whereas negative values indicate underestimation of discharge variance. Lines connect the T1 and T2 results for each model family.

**Figure S6.** Observed and one-step predicted monthly discharge for the selected univariate and multivariate MLP models under the T1 temporal configuration. Shaded areas represent 95% residual-based predictive intervals obtained using a 12-month moving-block bootstrap of pseudo-out-of-sample residuals generated exclusively within the calibration period. Gaps indicate missing observations or unavailable predictor combinations.

**Figure S7.** Multihorizon validation performance of the selected benchmark, linear regression, stochastic, and multilayer perceptron (MLP) models under the T1 and T2 temporal configurations. Panels show mean absolute error (MAE) and root mean square error (RMSE) at prediction horizons of 1, 3, 6, 12, and 24 months. Errors are expressed in m³ s⁻¹. Lines connect results from the same model family across horizons. Because the number of valid predictions varied among models and horizons, changes at longer horizons should be interpreted descriptively.

**Figure S8**. Mean monthly discharge during the pre-2012 (January 1966–December 2011) and post-2012 (January 2012–April 2019) periods. Error bars represent 95% confidence intervals.

**Figure S9.** Mean monthly climatological cycles of (A) precipitation and (B) river discharge over the common observation period. Blue lines and points represent monthly means, whereas shaded areas represent the mean ± one interannual standard deviation for each calendar month. Precipitation and discharge are expressed in mm and m³ s⁻¹, respectively.
